# Empirical approaches for rock burst prediction: A comprehensive review and application to the new level of El Teniente Mine, Chile

**DOI:** 10.1016/j.heliyon.2024.e26515

**Published:** 2024-02-20

**Authors:** Nayadeth Cortés, Amin Hekmatnejad, Pengzhi Pan, Ehsan Mohtarami, Alvaro Pena, Abbas Taheri, Cristian González

**Affiliations:** a Escuela de Ingenieria Quimica, Pontificia Universidad Católica de Valparaíso, Chile; b State Key Laboratory of Geomechanics and Geotechnical Engineering, Institute of Rock and Soil Mechanics, Chinese Academy of Sciences, Wuhan, 430071, China; c Department of Civil and Geomechanics Engineering, Arak University of Technology, Arak, Iran; d Escuela de Ingeniería de Construcción y Transporte, Pontificia Universidad Católica de Valparaíso, Chile; e Associate Professor in Geomechanics, Chair in Mine Design, The Robert M. Buchan Department of Mining, Queen's University, Kingston, Canada

## Abstract

Rockburst phenomena pose significant challenges in the mining industry, particularly with increased underground activities at greater depths. These sudden failures not only jeopardize personnel safety but also impact mining investments. Consequently, it becomes crucial to assess the reliability and effectiveness of empirical methods employed for predicting rock burst occurrences and their severity, an ongoing subject of debate within the scientific community. This research presents a comprehensive review of empirical approaches for rock burst prediction. Subsequently, these approaches are applied to predict rock burst occurrences and its intensity within sections of a tunnel at the new level of El Teniente mine in Chile. Most of these methods rely on single-factor criteria to predict the likelihood and severity of rock bursts. However, inconsistencies are observed in the results obtained from these approaches in numerous cases. This discrepancy highlights the influence of various input parameters on rock burst estimations and emphasizes that single-index criteria may not encompass all the pertinent factors that contribute to this phenomenon. Consequently, such criteria may inadequately estimate or reflect the probability of rock burst occurrences. Given the multifaceted nature of rock burst phenomena, which depend on multiple factors, it becomes imperative to explore new approaches that consider a broader range of influencing factors, thereby yielding more realistic results. Hence, continued research is essential to develop new methods that address this issue comprehensively and ensure the safety of the mining industry.

## Highlights

• Rockbursts in mining: challenges at greater depths.
• Reviewing empirical rockburst prediction methods.
• Applying methods in El Teniente mine, Chile.
• Inconsistencies in single-factor prediction.
• Need for new multi-factorial rockburst approaches.

## 1 Introduction

Over the years, the demand for mineral resources has led mining companies to deepen their underground operations in search of new mineralized bodies [1]. However, this has posed significant challenges for engineers, including rock mass instability [2,3]. As operations deepen, increased stresses in the rock mass can induce failure in the surrounding rock of the excavation [2,4,5]. Additionally, mining constructions can alter the pre-existing stress field, causing deformations in the mass and potential failures, depending on the magnitude of the induced stresses relative to the strength of the rock mass [6,7].

Rock mass instability can result in various failure mechanisms such as rock block failure, slabbing, spalling, and zonal disintegration [[8], [9], [10], [11]]. However, the most prominent phenomenon observed is rockburst, which frequently takes place in areas with hard or brittle rock formations and high levels of in-situ stresses [[12], [13], [14], [15], [16], [17], [18]]. Nonetheless, it should be noted that rockburst occurrences are not exclusively confined to these specific types of rock masses. Rockburst is recognized as a crucial failure mechanism in deep mines and tunnels [[19], [20], [21], [22]] and intensifies with increasing excavation depth [[23], [24], [25], [26]], leading to a rise in rockburst incidents worldwide [[27], [28], [29]], becoming a universal problem [18,30,31]. Common locations where rockburst incidents have occurred are in mines in South Africa [32], Chile [33], Canada [34], China [35], Western Australia [36], USA [37], Norway [38], Korea, Russia, Sweden [18].

Although a universally accepted definition for this failure mechanism, known as rockburst, has not yet been established [33], various studies have attempted to describe it, generating multiple perspectives on the matter [30]. To predict the occurrence of this phenomenon, various empirical methods have been proposed, ranging from theoretical to mathematical approaches, to successfully assess the potential of this phenomenon. However, the complex characteristics of rockburst pose a significant challenge for the global mining industry [16,39], due to the lack of a universally accepted method that can predict the timing and location of rockburst occurrences. Instead, it only identifies high probability zones of rockburst based on empirical criteria, numerical models, or personal experience [30], considering key factors [40,41].

The prediction methods proposed by various researchers indicate that rockburst is influenced by inherent rock properties (strength and brittleness), the external environment (stress conditions), dynamic disturbances, geological structures [42], excavation sequence, and the depth at which the excavation occurs [16,40,41]. For this reason, many studies have focused on investigating the influence of these factors on this phenomenon [34,[43], [44], [45]]. One of the factors influencing rockburst occurrence is the presence of geological structures such as joints and faults, which can cause a significant increase in stresses [46]. Therefore, it is more likely for this phenomenon to occur when such structures are close to the excavation [45,[47], [48], [49], [50], [51]]. Another factor is the high in-situ stresses present in the external environment, which are the primary cause of deformation energy accumulation [52].

Rockburst is a phenomenon that causes serious incidents in underground mining, endangering people, equipment, and galleries, leading to delays and disruptions in mining operations, and causing significant consequences that can significantly impact the long-term economic outcomes of the mining company [7,18,45,[53], [54], [55], [56], [57]]. Therefore, the reliability and effectiveness of these systems remain a subject of study and debate in the scientific community, as it focuses on analyzing the accuracy and reliability of empirical methods that predict the occurrence of rockburst and its severity. This involves evaluating the ability of criteria to consider multiple factors that can influence rock stability and determine the likelihood of this phenomenon occurring.

The objective of this research is to make a substantial contribution towards a more rigorous evaluation of the reliability of widely used empirical methods in underground construction for predicting rockburst occurrences. To achieve this goal, an extensive literature review will be conducted, accompanied by a comprehensive analysis of the results from previous research. Additionally, each criterion will be applied to the case study of the New Level of El Teniente Mine in Chile, enabling a thorough assessment of its effectiveness. By combining insights from literature review, analysis of previous research, and the specific case study, this research aims to enhance our understanding and evaluation of these empirical methods in predicting rockburst occurrences accurately and reliably.

## 2 Definition and classification of rock burst

So far, there is no universally accepted definition for the rock burst phenomenon [30]. Over the years, various perspectives have been proposed from 1946 to 2021, encompassing concepts such as deformation energy, highly stressed zones, brittle or massive rocks, violent and sudden ejection, occurrence at great depths, among others. However, Jiang et al. [58] presents a simple definition of rock burst as the release of elastic energy in the rock mass due to an increase in tangential stress and a decrease in radial stress. Table 1 provides details of the most recognized authors along with their respective proposed definitions for rock burst.

**Table 1:** Definitions of rockburst according to various authors.

Auhtor	Definition
Terzaghi [59]	Hard and brittle rocks experiencing high stress can cause abrupt separation or falling in tunnel walls.
Cook [4]	It is considered a stability issue that occurs outside the blasting time, and for a violent rock fracture to occur, additional energy is required in addition to the energy stored in the form of deformation in the sample
Cook et al. [12]	It is a violent and uncontrolled disruption of the rock, characterized by an intense release of energy caused by the falling of rock fragments. As the depth increases, the rate of energy release also increases, implying a higher risk. However, this risk can be reduced through methods that allow for a more controlled and non-violent dissipation of energy.
Hoek and Brown [5]	Present in deep mines, illustrating the phenomena of explosive brittle fracture.
Ortlepp and Stacey [60]	Damage produced in the tunnel as a result of a seismic event or closely related to it. There are no restrictions regarding the magnitude of this event if it generates sufficient energy to cause significant damage. As depths increase and tunnel construction areas become more challenging, the probability of rock burst occurring also increases. Furthermore, rock excavations carried out with machinery can also increase the chances of rock burst.
Kaiser et al. [34]	Violent and sudden damages that occur in underground excavations and are associated with seismic events. It can occur in rocks subjected to high stresses, whether they are massive or jointed, when the stresses exceed the strength and cause structural failures.
Kaiser et al. [2]	Violent failures occurring in areas where hard, jointed rocks are being extracted, resulting in immediate or violent damage to the excavation. While a rockburst may be associated with a seismic event, it is not directly caused by the seismic event itself.
He et al. [7]	Caused by the excessive stress present in the massif or in intact brittle rocks when the stress exceeds the local compressive strength of the material. All rockbursts generate seismic waves and disturbances that are recorded by seismological stations.
Zhao et al. [61]	Hazards that occur in hard and brittle rock masses under conditions of high in-situ stresses or high stress concentrations. This geomechanical risk is frequent and significant, especially in massive and brittle rocks located at depths exceeding 1000 m.
Meng et al. [16]	Originating violently or suddenly, always associated with a seismic event. During a rock burst, a large amount of energy is released, causing the ejection of rock blocks from the adjacent rock mass.

Although there are different approaches to defining rockburst, it has been established that this instability is related to the sudden release of elastic energy from the rock mass due to disturbances in the in-situ stresses during underground construction [12,18]. However, the precise mechanism that triggers rockburst is still not fully understood [55,62].

**Fig. 1:**
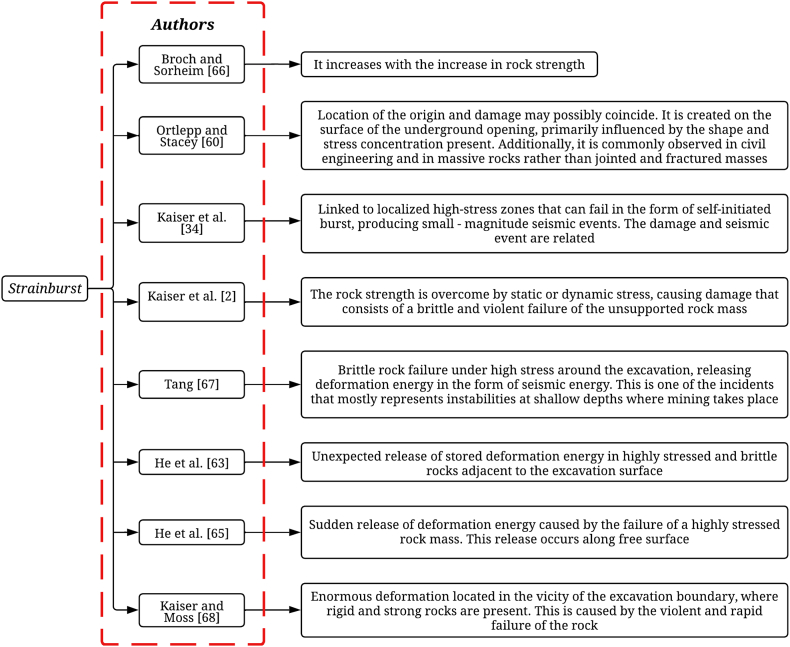
Definition of strainburst according to various authors.

**Fig. 2:**
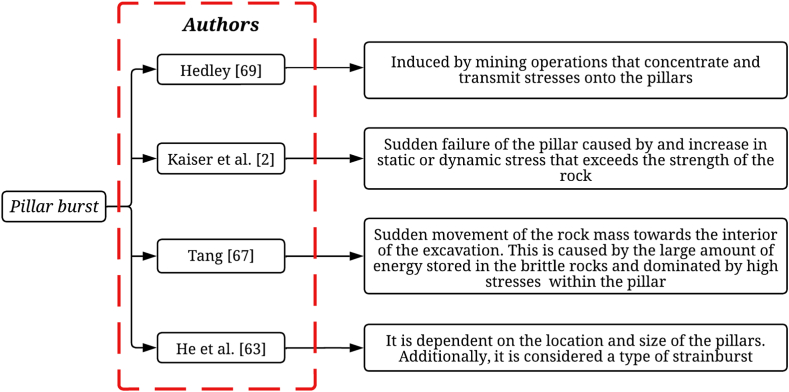
Definition of pillar burst according to various authors.

**Fig. 3:**
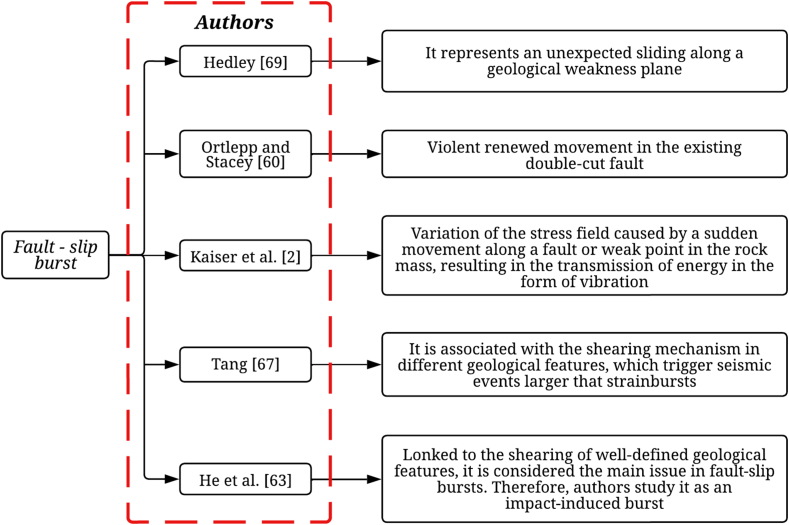
Definition of fault-slip burst according to various authors.

Kaiser and Cai [41] emphasize the importance of a specific and unified classification of rockburst for proper selection of support or control measures. In this regard, numerous researchers have identified that this instability is generally classified into three main categories: strainburst, pillar burst, and fault-slip burst, based on the manifested failure mechanism [2,[63], [64], [65]]. These primary types of rockburst are widely recognized in the mining industry and have been defined by various experts in geomechanics, as shown in Fig. 1, Fig. 2, Fig. 3.

During the classification of rockburst, their suitability has been questioned from an engineering perspective. According to Kaiser et al. [2], this classification combines causes and effects in a way that is not appropriate, as it often involves a measure of severity that is not relevant from the excavation design point of view. Instead, the authors propose classifying rockburst based on their triggering mechanism, damage mechanism, and severity, which is more suitable for the respective tunnel construction.

## 3 Literature review

Predicting rock bursting, a critical safety concern in underground mining and tunneling, involves various methods, each with its unique application and insights. Empirical methods, based on historical data and observations, use indices to assess risk factors such as rock strength, mining depth, and stress conditions [5,38,64,[66], [67], [68], [69], [70], [71], [72], [73]]. While straightforward, these methods may not be universally applicable due to their reliance on specific case studies.

Numerical modeling offers a more customized approach. Techniques like Finite Element Analysis (FEA), Discrete Element Method (DEM) [[74], [75], [76], [77], [78], [79]] simulate the physical and mechanical behavior of rock masses under stress. This detailed analysis aids in understanding stress distribution and potential failure zones, tailoring predictions to the specific mining environment.

Geophysical monitoring plays a pivotal role in real-time assessment. Utilizing tools like microseismic systems [15], acoustic emission sensors [[80], [81], [82]], and ground-penetrating radar, this approach detects early signs of rock bursts, such as seismic activity or stress pattern changes. It is especially valuable for immediate risk assessment and mitigation.

The emergence of machine learning and data analytics has introduced a dynamic element to rock burst prediction. Analyzing extensive data from seismic activities, geological information, and induced stresses, these advanced algorithms can uncover patterns not evident through traditional methods. Machine learning models, trained with various input parameters, offer adaptive and evolving predictions [[83], [84], [85]].

Laboratory testing complements these approaches by replicating rock burst conditions on a smaller scale. Tests like triaxial compression help in understanding the rock's mechanical behavior under high-stress conditions, although translating these findings to full-scale scenarios remains challenging [[86], [87], [88], [89], [90], [91], [92]].

An integrated approach, blending multiple methods, often results in the most accurate predictions. For instance, combining numerical modeling with empirical data and real-time monitoring can enhance the reliability of predictions, offering a comprehensive strategy for managing rock burst risks in mining operations. This multifaceted approach leverages the strengths of each method, addressing the complexity and variability inherent in predicting rock bursts.

Empirical approaches for predicting rock bursts have become a significant area of study in geotechnical engineering, particularly for assessing the safety and feasibility of underground excavations. The work of Zhou et al. [30] and others highlights the evolution of these methods, emphasizing their importance in preliminary excavation design and risk assessment. While empirical methods offer practical tools for predicting rock bursts, it's crucial to acknowledge, as Zhao et al. [61] and Askaripour et al. [18] have, that these approaches are continuously refined and validated, and may sometimes yield inconsistent results. The classification of empirical rockburst prediction methods into two categories – single-index criteria and multiple-factor evaluation criteria – provides a clearer framework for understanding these approaches. According to Zhou et al. [30] and Qiu and Feng [93], the single-index criterion revolves around fundamental theories like brittleness, stress/resistance, stiffness, and energy of the rock. On the other hand, the multiple-factor evaluation criteria consider a broader range of parameters, such as rock mass quality, failure probability, stress conditions, local stiffness, and support systems, as elaborated by Kaiser et al. [94]. A notable aspect of these empirical methods is their focus on the interplay between stress and the uniaxial compressive strength of the rock, which is a common theme among many researchers in the field. The approach by Qiu and Feng [93] stands out in this regard as a comprehensive multiple-factor criterion used in contemporary research. This methodology not only takes into account a variety of influential factors but also reflects the complex nature of rockburst phenomena in underground settings. It is evident that empirical methods provide a foundational basis for rockburst prediction. However, the diversity in these methods and their varying degrees of focus on different parameters underscore the complexity of accurately predicting rock bursts. The ongoing development and refinement of these methods are essential in enhancing their reliability and applicability in diverse geological settings. Therefore, it is crucial to continue exploring these empirical approaches, considering their limitations and potential for improvement in the context of evolving understanding of rock mechanics and underground engineering challenges.

When evaluating the strengths and weaknesses of empirical approaches for predicting rock bursts, a balanced perspective is crucial. The strengths lie in their practicality and ease of use, making them accessible for rapid assessments and preliminary analyses. They are grounded in historical data, which provides a solid empirical basis for predictions and helps in understanding past events. Additionally, their adaptability allows these methods to be adjusted to suit specific mining conditions. On the flip side, empirical approaches have limitations in predictive power, especially in conditions significantly different from past scenarios. This limitation stems from their reliance on historical data, potentially leading to inconsistencies in predictions. Different empirical methods can yield varying results for the same scenario, highlighting the diverse factors considered by each. Moreover, these methods lack dynamism, as they may not effectively account for real-time changes in mining conditions, such as evolving stress patterns or new geological discoveries. This static nature can limit their effectiveness in rapidly changing or unexplored environments. The subsequent sections of the paper will delve into a more detailed analysis of each approach's pros and cons, offering a comprehensive understanding of their applicability and limitations in the context of rock burst prediction.

## 4 Methodology

**Fig. 4:**
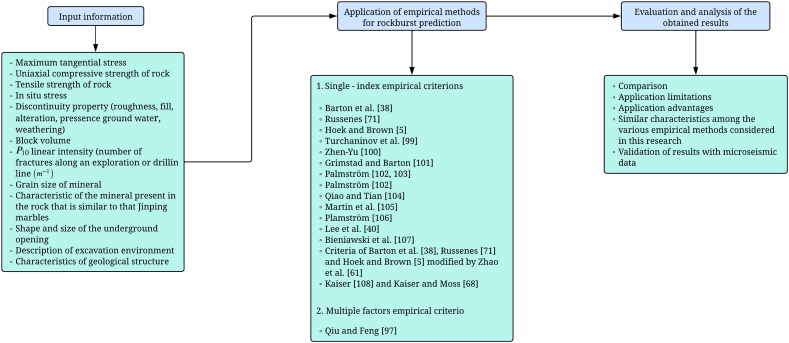
Methodology used in the research.

A comprehensive assessment of the feasibility and applicability of various empirical criteria for predicting rockburst and its severity was carried out in 54 sections of a tunnel at New El Teniente Mine, Chile. The procedures and criteria used to determine rockburst and its corresponding severity are described. It is used the micro seismic data set to verify the obtained results of these empirical approaches and observations at the filed. During this process, the specific advantages and limitations of each criterion were analyzed, allowing for a thorough comparison. It is important to note that all these empirical methods were applied assuming quasi-static conditions. It is worth mentioning that the selection of these approaches for our study was guided by the compatibility of the available data sets with the evaluation requirements of these empirical methods. This criterion ensures that the chosen approaches are well-suited to the data we have, allowing for a more accurate and meaningful analysis of their effectiveness. The details of each step carried out in this research can be observed in Fig. 4.

## 5 Empirical rock burst prediction approach

**Fig. 5:**
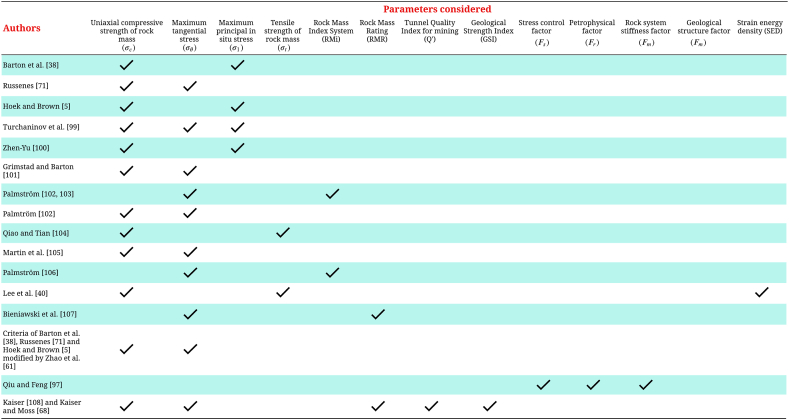
Authors studied in the research with their respective parameters used to predict rockburst.

In the subsequent sections, we present the empirical approaches utilized in this study (Fig. 5). Each method is detailed along with its corresponding parameters, and an analysis of its advantages and disadvantages is provided, drawing on current state-of-the-art knowledge. This approach ensures a thorough understanding of the selected methodologies and their applicability to our research objectives.

### 5.1 Barton et al. [38]

According to the authors, the rock burst condition originates due to stress problems in competent rock. Therefore, the utilization of equations (1), (2)) is proposed to determine the stress condition present in the rock, which are classified in Table 2.

(1) $$\frac{\sigma_{c}}{\sigma_{1}}$$

**Table 2:** Classification of stress levels in relation to stress-strength after Barton et al. [38].

Criteria		Classification
$\frac{\sigma_{c}}{\sigma_{1}}<2.5$	$\frac{\sigma_{t}}{\sigma_{1}}<0.16$	Heavy rockburst (massive rock)
$\frac{\sigma_{c}}{\sigma_{1}}=2.5-5$	$\frac{\sigma_{t}}{\sigma_{1}}=0.16-0.33$	Mild rockburst (massive rock)
$\frac{\sigma_{c}}{\sigma_{1}}=5-10$	$\frac{\sigma_{t}}{\sigma_{1}}=0.33-0.66$	High stress, very tight structure
$\frac{\sigma_{c}}{\sigma_{1}}=10-200$	$\frac{\sigma_{t}}{\sigma_{1}}=0.66-13$	Medium stress
$\frac{\sigma_{c}}{\sigma_{1}}>200$	$\frac{\sigma_{t}}{\sigma_{1}}>13$	Low stress, near surface

Or in terms of the tensile strength of the rock

(2) $$\frac{\sigma_{t}}{\sigma_{1}}$$

where.

$\sigma_{1}$ = Maximum in-situ principal stress
$\sigma_{c}$ = Uniaxial compressive strength of the rock
$\sigma_{t}$ = Tensile Strength of the rock

It is worth to mention that Equations (1), (2)) are Stress reduction factor (SRF) in the Q system, which assigns scores to faults, strength relationships, and stresses in hard rocks, as well as the squeezing or swelling phenomenon, which measures the loosening load in underground constructions developed in cut areas and clayey rocks. It also considers the stresses in competent rock and the compressive loads or squeezing in incompetent plastic rocks. According to the authors, the rockburst condition originates due to stress problems in competent rock. Therefore, the utilization of equations (1), (2)) is proposed to determine the stress condition present in the rock, which are classified in Table 2.

The evaluation method presents various advantages and limitations that are mentioned in Table 3.

**Table 3:** Advantages and limitations present in the prediction method proposed by Barton et al. [38].

Advantage	Limitations
Simple mathematical equation.	It considered the uniaxial compressive strength of the rock, which is considered a standard strength that leads to very conservative analyses [95].
Easily obtainable input factors.	The application of SRF is not clear for rockburst, buckling y/o squeezing [[96], [97], [98]].
Quantitative form of predicting rockburst, thus reducing subjectivity and feasibility of qualitative assessment.	It applies the maximum in-situ stress, despite being unreasonable to apply in prediction criteria, as according to Kirsch [99], $\sigma_{\theta }$ progressively decreases from $3\sigma_{1}-\sigma_{3}$ to $\sigma_{1}$ with distance from the tunnel. Additionally, the resulting ${\sigma_{\theta }}^{\max}$ is influenced by excavation-induced stresses [65].
Quick calculation.	The values 5 and 10, or 0.66 and 0.33, are not clearly considered within a single classification range.
Classification and numerical method developed based on the analysis of observations recorded by a researcher.	It uses the tensile Strength factor of the rock, which is of little importance because the in-situ stresses at depth are never in tension but in compression [2,42].
The base case includes various rock types, allowing the criterion to be applied without inconvenience.	It does not consider the properties of the discontinuities and structurally controlled failures. It assumes that rock burst only occurs in a competent and massive rock.
	It does not consider the anisotropy or heterogeneity property of the rock [[100], [101], [102]].
	When considering the uniaxial compressive Strength of the rock, the scale effect is overlooked, which considers the heterogeneity of the rock, such as microfractures and grains of different mineral compositions, when it comes to rock and rock mass strength. The existence of this scale effect on strength has been demonstrated by various author, such as [5,[103], [104], [105], [106], [107]].

### 5.2 Russenes [67]

The author conducted a study on rockburst cases in tunnels in the steep slopes of Norway. In this area, various types of rockburst activities were observed, which were classified into four classes, ranging from 0 (not rockburst) to 3 (high or intense rockburst activity). These cases were evaluated by comparing the values of strength and maximum tangential stress obtained from point load tests and finite element models, and from the equation proposed by Kirsch [99], respectively. Based on these results, the author proposes a criterion that considers the properties of the rock and the state of in situ stresses, as shown in Equations (3), (4)) and classified in Table 4.

(3) $$\frac{I_{s}}{\sigma_{\theta }}$$

**Table 4:** Classification of rockburst levels after Russenes [67].

Criteria		Classification
$\frac{\sigma_{\theta }}{\sigma_{c}}<0.2$	$\frac{I_{s}}{\sigma_{\theta }}\;>0.2$	No rockburst
$0.2\leq \frac{\sigma_{\theta }}{\sigma_{c}}<0.3$	$\frac{I_{s}}{\sigma_{\theta }}=0.2-0.15$	Light rockburst
$0.3\leq \frac{\sigma_{\theta }}{\sigma_{c}}<0.55$	$\frac{I_{s}}{\sigma_{\theta }}=0.15-0.083$	Moderate rockburst
$\frac{\sigma_{\theta }}{\sigma_{c}}\geq 0.55$	$\frac{I_{s}}{\sigma_{\theta }}\;<0.083$	Strong or violent rockburst

It can be expressed as

(4) $$\frac{\sigma_{\theta }}{\sigma_{c}}$$

where.

$\sigma_{\theta }$ = Maximum tangential stress present around the tunnel

$\sigma_{c}$ = Uniaxial compressive strength of the rock

$I_{s}$ = Rock's point load strength

Table 5 presents the advantages and limitations of the criterion proposed by Russenes [67].

**Table 5:** Advantages and limitations present in the prediction method proposed by Russenes [67].

Advantage	Limitations
Simple mathematical equation	It considered the uniaxial compressive strength of the rock, which is considered a standard strength that leads to very conservative analyses [95].
Easily obtainable input factors	Does not mention the direction of the maximum tangential stress
Quantitative form of predicting rockburst, thus reducing subjectivity and feasibility of qualitative assessment.	The value 0.15 is not clearly considered within a single classification range of the $\frac{I_{s}}{\sigma_{\theta }}$ criterion.
Quick calculation	Insufficient lithological consideration is presented [30].
Classification developed based on the analysis of a real case	It does not consider the properties of the discontinuities.
	It does not consider the anisotropy or heterogeneity property of the rock
	It estimates the maximum tangential stress based on Kirsch's equation [99], which is only applicable to circular excavations.
	When considering the uniaxial compressive Strength of the rock, the scale effect is overlooked, which considers the heterogeneity of the rock, such as microfractures and grains of different mineral compositions, when it comes to rock and rock mass strength. The existence of this scale effect on strength has been demonstrated by various author, such as [5,[103], [104], [105], [106], [107]].
	It estimates that any type of failure occurring in the rock mass (ejection, spalling, or fracture face) is considered a rockburst.
	Uniaxial compressive strength of the rock and Rock's point load strength are highly correlated through direct linear equations.

### 5.3 Hoek and Brown [5]

An analysis of various observation records in deep gold mines in South Africa was conducted, which showed rock failures around square tunnels in areas of massive quartzite. These mines exhibit an in-situ stress ratio of k = 0.5, and the properties of the massive quartzite show very good quality. The records were graphically represented using the values of the in situ vertical stress at which the lateral wall of the tunnel collapses. Based on this, the author developed the classification presented in Table 6 using the prediction criterion described in Equation (5). Additionally, Table 7 mentions some advantages and disadvantages associated with this empirical method.

(5) $$\frac{\sigma_{1}}{\sigma_{c}}$$

where.

**Table 6:** Classification of rockburst levels after Martin et al. [108].

Criteria	Classification
$\frac{\sigma_{1}}{\sigma_{c}}\leq 0.1$	Stable opening without support. No damages are observed
$\frac{\sigma_{1}}{\sigma_{c}}=0.2$	Minor spalling requiring light support
$\frac{\sigma_{1}}{\sigma_{c}}=0.3$	Severe spalling requiring moderate support
$\frac{\sigma_{1}}{\sigma_{c}}=0.4$	Heavy support requirement for the opening to be stable. Very severe spalling
$\frac{\sigma_{1}}{\sigma_{c}}>0.5$	Possible rockburst condition

**Table 7:** Advantages and limitations present in the prediction method proposed by Hoek and Brown [5].

Advantage	Limitations
Simple mathematical equation.	It considered the uniaxial compressive strength of the rock, which is considered a standard strength that leads to very conservative analyses [95].
Easily obtainable input factors.	Applied only for an in-situ stress ratio of 0.5.
Quantitative form of predicting rockburst, thus reducing subjectivity and feasibility of qualitative assessment.	It applies the maximum in-situ stress, despite being unreasonable to apply in prediction criteria, as according to Kirsch [99], $\sigma_{\theta }$ progressively decreases from $3\sigma_{1}-\sigma_{3}$ to $\sigma_{1}$ with distance from the tunnel. Additionally, the resulting ${\sigma_{\theta }}^{\max}$ is influenced by excavation-induced stresses [65].
Quick calculation.	It does not provide a range of values in all classifications.
Classification developed based on the analysis of several real-life cases.	It does not consider the properties of the discontinuities.
	When considering the uniaxial compressive Strength of the rock, the scale effect is overlooked, which considers the heterogeneity of the rock, such as microfractures and grains of different mineral compositions, when it comes to rock and rock mass strength. The existence of this scale effect on strength has been demonstrated by various author, such as [5,[103], [104], [105], [106], [107]].
	It does not consider the anisotropy or heterogeneity property of the rock.
	It estimates that rockburst only occurs in one type of competent and massive rock, without considering the probability of rockburst occurrence in blocky rocks.

$\sigma_{1}$ = Maximum in-situ principal stress

$\sigma_{c}$ = Uniaxial compressive strength of the rock

### 5.4 Turchaninov et al. [109]

Turchaninov et al. [66] investigated the stress state within the Earth's crust caused by gravitational forces and tectonic stresses. This study demonstrated that the stress state is not uniform, concluding that the directions and magnitudes of principal stresses vary from one region to another. Furthermore, when examining the stresses present in the Kola Peninsula, it was observed that the most significant compressive stresses occur in the horizontal plane, increasing with depth. For this reason, in 1981, Turchaninov et al. decided to investigate the effect and influence of tectonic forces on the design of underground structures.

The research conducted by Turchaninov et al. [109] reveals that rock masses subjected to tectonic stresses can trigger destructive processes in the surrounding rock around an excavation. These processes can manifest in the form of rockburst, natural bursting, or spalling of the rock during excavation construction. To identify the possibility of occurrence of these instabilities, the authors propose a numerical criterion that classifies the intensity of rockburst. This method is based on equation (6) and is classified in Table 8, according to the range of values and the degree of rock crushing.

(6) $$\frac{\left|\sigma_{\theta }+\sigma_{L}\right|}{\sigma_{c}}$$

where.

**Table 8:** Classification of rockburst levels after Turchaninov et al. [109].

Criteria	Classification
$\frac{\sigma_{\theta }+\sigma_{L}}{\sigma_{c}}<0.3$	Stable excavation. No rockburst occurrence.
$0.3\leq \frac{\sigma_{\theta }+\sigma_{L}}{\sigma_{c}}<0.5$	Gradual flaking and peeling-induced damage. Probable rockburst occurrence.
$0.5<\frac{\sigma_{\theta }+\sigma_{L}}{\sigma_{c}}\leq 0.8$	Destruction occurring at a constant rate, reaching its limit in rockbursts and outbursts. Highly probable rockburst occurrence.
$\frac{\sigma_{\theta }+\sigma_{L}}{\sigma_{c}}>0.8$	Massive destruction accompanied by a rockburst. Severe and violent rockburst.

$\sigma_{\theta }$ = Tangential stress near the boundary, perpendicular to the longitudinal axis (maximum tangential stress).

$\sigma_{L}$ = Longitudinal stress near the boundary, parallel to the longitudinal axis of the pit

$\sigma_{c}$ = Uniaxial compressive strength of the rock

This numerical method offers several advantages and limitations, which are mentioned in Table 9.

**Table 9:** Advantages and limitations present in the prediction method proposed by Turchaninov et al. [109].

Advantage	Limitations
Simple mathematical equation.	It considered the uniaxial compressive strength of the rock, which is considered a standard strength that leads to very conservative analyses [95].
Easily obtainable input factors.	The proposed equation is only applicable to zones where horizontal stresses exceed vertical stresses (reverse stress regime).
Quantitative form of predicting rockburst, thus reducing subjectivity and feasibility of qualitative assessment.	The stresses affecting the stability of the excavation are overestimated by summing the longitudinal and maximum tangential stresses at the boundary of the opening.
Quick calculation	It indicates the stress directions with respect to a pit, not to an underground opening. This can cause confusion among different authors who apply this numerical method.
It indicates the direction of the maximum tangential stress.	It does not consider the properties of the discontinuities.
Classification developed based on the analysis of several real-life cases.	When considering the uniaxial compressive Strength of the rock, the scale effect is overlooked, which considers the heterogeneity of the rock, such as microfractures and grains of different mineral compositions, when it comes to rock and rock mass strength. The existence of this scale effect on strength has been demonstrated by various author, such as [5,[103], [104], [105], [106], [107]].
It considers that the formation of the failure starts when the stress at the excavation boundary is 0.3 times the uniaxial compressive strength. This relationship is supported by various researchers through laboratory tests [107].	It estimates that rockburst only occurs in one type of competent and massive rock, without considering the probability of rockburst occurrence in blocky rocks.
	It does not consider the anisotropy or heterogeneity property of the rock.

### 5.5 Zhen-yu [110]

It is proposed that the physical basis for the occurrence of rockburst is the generation and accumulation of stresses induced by underground excavation. These stresses trigger the formation of cracks adjacent to the rock mass surface, following clearly defined directions. Based on this physical basis, the author proposes and determines the rockburst activity index through the observation of underground excavations in China. In this way, the classification presented in Table 10 is established based on Equation (7).

(7) $$\frac{R_{c}}{\sigma_{1}}$$

where.

**Table 10:** Classification of rockburst levels after Zhen-Yu [110].

Criteria	Classification
$\frac{R_{c}}{\sigma_{1}}<2.5$	High rockbursting activity and very strong cracking noises
$2.5<\frac{R_{c}}{\sigma_{1}}<5.5$	Moderate rockbursting activity and strong cracking noises
$5.5<\frac{R_{c}}{\sigma_{1}}<13.5$	Low rockbursting activity and light noises
$\frac{R_{c}}{\sigma_{1}}>13.5$	No rockbursting. No noises

$\sigma_{1}$ = Maximum in-situ principal stress

$R_{c}$ = Uniaxial compressive strength of the rock

The advantages and limitations of this criterion are presented in Table 11.

**Table 11:** Advantages and limitations present in the prediction method proposed by Zhen-Yu [110].

Advantage	Limitations
Simple mathematical equation.	It considered the uniaxial compressive strength of the rock, which is considered a standard strength that leads to very conservative analyses [95].
Quantitative form of predicting rockburst, thus reducing subjectivity and feasibility of qualitative assessment.	It applies the maximum in-situ stress, despite being unreasonable to apply in prediction criteria, as according to Kirsch [99], $\sigma_{\theta }$ progressively decreases from $3\sigma_{1}-\sigma_{3}$ to $\sigma_{1}$ with distance from the tunnel. Additionally, the resulting ${\sigma_{\theta }}^{\max}$ is influenced by excavation-induced stresses [65].
Easily obtainable input factors.	The proposed equation is only applicable to zones where horizontal stresses exceed vertical stresses (reverse stress regime).
Quick calculation.	The base case only considers the behavior of a circular excavation.
Classification developed based on the analysis of several real-life cases.	It studies the stress concentration coefficient (k) based on the relationship between the maximum tangential stress and the major principal stress.
	It does not consider the properties of the discontinuities.
	When considering the uniaxial compressive Strength of the rock, the scale effect is overlooked, which considers the heterogeneity of the rock, such as microfractures and grains of different mineral compositions, when it comes to rock and rock mass strength. The existence of this scale effect on strength has been demonstrated by various author, such as [5,[103], [104], [105], [106], [107]].
	It does not consider the anisotropy or heterogeneity property of the rock.
	The values 13.5, 5.5, and 2.5 are not considered within any range of the classification.
	It estimates that rockburst only occurs in one type of competent and massive rock, without considering the probability of rockburst occurrence in blocky rocks.

### 5.6 Grimstad and Barton [111]

An update of the Q-system was carried out using 1050 new cases of road tunnels constructed in the last 10 years, covering a wide range of rock quality from exceptionally poor to extremely good. The parameters considered were the same as proposed by Barton et al. [38], but modifications were made to the classifications of the Support Resistance Factor (SRF). This was because hard and massive rock, subjected to high stresses, requires a higher level of support than suggested by the corresponding Q-value. Therefore, the authors demonstrated that the SRF value should be increased from 20 to 400 in extreme cases of massive rock found in high-stress zones. To achieve this, adjustments were made to the relationship between the maximum tangential stress ($\sigma_{\theta }$) and the compressive strength of the rock ($\sigma_{c}$), as well as between $\sigma_{c}$ and the maximum principal stress ($\sigma_{1}$), based on eight selected cases out of the 1050 considered in the update. The new classification ranges are presented in Table 12, while Table 13 lists the various advantages and limitations of this updated criterion.

**Table 12:** Classification of rockburst levels after Grimstad and Barton [111].

Criteria		Classification
$\frac{\sigma_{c}}{\sigma_{1}}<2$	$\frac{\sigma_{\theta }}{\sigma_{c}}<1$	Heavy rockburst (strainburst) and immediate dynamic deformations in massive rock
$\frac{\sigma_{c}}{\sigma_{1}}=2-3$	$\frac{\sigma_{\theta }}{\sigma_{c}}=1-0.65$	Slabbing and rockburst after minutes in massive rock
$\frac{\sigma_{c}}{\sigma_{1}}=3-5$	$\frac{\sigma_{\theta }}{\sigma_{c}}=0.65-0.5$	Moderate slabbing after >1 h in massive rock
$\frac{\sigma_{c}}{\sigma_{1}}=5-10$	$\frac{\sigma_{\theta }}{\sigma_{c}}=0.4-0.3$	High stress, very tight structure. Usually favourable to stability, maybe unfavorable to wall stability
$\frac{\sigma_{c}}{\sigma_{1}}=10-200$	$\frac{\sigma_{\theta }}{\sigma_{c}}=0.3-0.01$	Medium stress, favourable stress condition
$\frac{\sigma_{c}}{\sigma_{1}}>200$	$\frac{\sigma_{\theta }}{\sigma_{c}}>0.01$	Low stress, near Surface, open joints

**Table 13:** Advantages and limitations present in the prediction method proposed by Grimstad and Barton [111].

Advantage	Limitations
Simple mathematical equation.	It considered the uniaxial compressive strength of the rock, which is considered a standard strength that leads to very conservative analyses [95].
Easily obtainable input factors.	The application of SRF is not clear for rockburst, buckling, and/or squeezing conditions [97].
Quantitative form of predicting rockburst, thus reducing subjectivity and feasibility of qualitative assessment.	It applies the maximum in-situ stress, despite being unreasonable to apply in prediction criteria, as according to Kirsch [99], $\sigma_{\theta }$ progressively decreases from $3\sigma_{1}-\sigma_{3}$ to $\sigma_{1}$ with distance from the tunnel. Additionally, the resulting ${\sigma_{\theta }}^{\max}$ is influenced by excavation-induced stresses [65].
Quick calculation.	The values 3, 5, and 10 or 0.65 and 0.3 are not clearly considered within a single classification range.
Classification and numerical method developed based on the analysis of observations recorded by a researcher.	There is no clear classification for values between 0.5 and 0.4 when evaluating the $\frac{\sigma_{\theta }}{\sigma_{c}}$ ratio.
Adds new tunnel observations for the modification of the criterion.	It does not consider the properties of the discontinuities.
Modifies the value ranges of the classification to ensure coherence between the support demand and the corresponding Q value.	It does not consider the anisotropy or heterogeneity property of the rock.
	When considering the uniaxial compressive Strength of the rock, the scale effect is overlooked, which considers the heterogeneity of the rock, such as microfractures and grains of different mineral compositions, when it comes to rock and rock mass strength. The existence of this scale effect on strength has been demonstrated by various author, such as [5,[103], [104], [105], [106], [107]].
	Does not mention the direction of the maximum tangential stress.
	It estimates that rockburst only occurs in one type of competent and massive rock, without considering the probability of rockburst occurrence in blocky rocks.

### 5.7 Palmström [112,113]

**Fig. 6:**
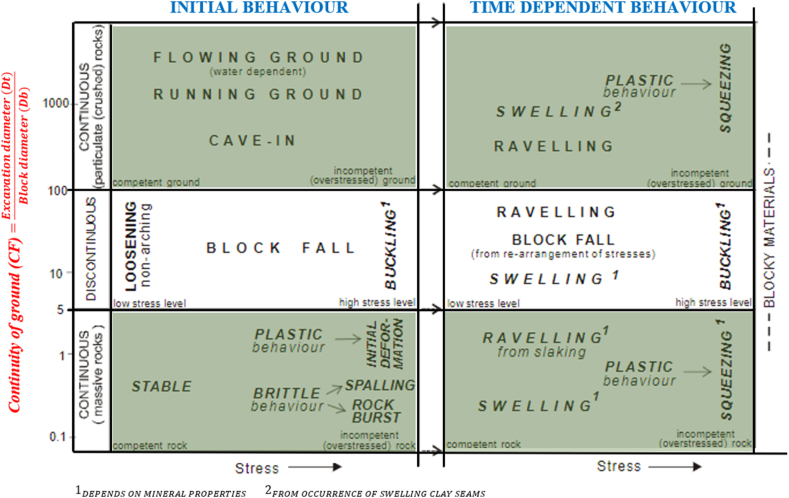
Behavior and instability of the rock mass, determined by stress conditions and ground continuity according to Palmström (2000).

The Rock Mass Index (RMi) is proposed as a method to characterize the strength of the rock mass, analyze its stability, and estimate the support requirements in underground excavations. This system can be used in continuous rock masses and also in the analysis of instabilities caused by overstressing in massive or intact rocks (rockburst conditions). To do so, it is necessary to characterize the continuity of the terrain to apply the appropriate stability analysis method for the specific rock mass under study. This is achieved through the continuity factor (CF), which is defined in equation (8) and classified in Fig. 6.

(8) $$\text{CF}=\frac{\text{Dt}}{\text{Db}}$$

where.

Dt = Excavation diameter or span, in metre

Db = Equivalent block diameter, in metre ($\text{Db}=\sqrt[{3}]{\text{Vb}}$, where Vb the block volume, measured in $m^{3}$)

In continuous terrains, where the continuity factor (CF) is less than approximately 5 (massive rock) and greater than approximately 100 (highly jointed rock), the main behavior of the rock mass is influenced by the stresses in the underground excavation. Therefore, the assessment of tunnel stability and failure mode is estimated using the competence factor (Cg), which is expressed in equation (9).

(9) $$\text{Cg}=\frac{\text{RMi}}{\sigma_{\theta }}$$

where.

The Rock Mass Index (RMi) is calculated for different types of rock masses—discontinuous and massive—using Equations (10), (11)), respectively. This index serves as a quantitative measure to assess the characteristics of rock masses based on their structure.

• Jointed rock

(10) $$\text{RMi}=\sigma_{c}\times \text{JP}=\sigma_{c}\times 0.2\sqrt{\text{jC}}\times {\text{Vb}}^{D}\;\left(D=0.37\times {\text{jC}}^{-0.2}\right)$$

• Massive rock

(11) $$\text{RMi}=\sigma_{c}\times f_{\sigma }=\sigma_{c}\times {\left(0.05/\text{Db}\right)\;}^{0.2}$$

The symbols represent:

JP = Jointing parameter, is a measure of the intensity of jointing given as the block size ($\text{JP}=0.2\sqrt{\text{jC}}\times {\text{Vb}}^{D};\text{in}\;\text{massive}\;\text{rock}\;\text{JP}=1$)

jC = Joint condition factor, which combines the parameters of joint size (jL), joint roughness (jR), and joint alteration (jA) using the equation jC=jL×jR/jA

Vb = Block volumen, measured in $m^{3}$

$\sigma_{c}$ = Uniaxial compressive strength of intact rock (MPa).

$f_{\sigma }$ = Massivity parameter it is the scaling for compressive strength, given as $f_{\sigma }={\left(0.05/\text{Db}\right)}^{0.2}$ where Db is the block diameter, measured in meters

$\sigma_{\theta }$ = Tangential stress in the rock masses can be derived from either the vertical or horizontal stress of the rock.

When Cg is greater than 1, the ground is considered competent, with stresses around the excavation that do not exceed the strength of the rock (stable rock). On the other hand, when Cg is less than 1, it indicates an incompetent rock mass that can lead to various instability modes induced by stresses. In this latter case, different failure modes are observed in brittle rock masses, such as rockburst and spalling. These modes are classified according to the value of Cg and are presented in Table 14.

**Table 14:** Classification of the competence factor after Palmström [112].

Criteria	Classification
Cg<0.5	Heavy rockburst
Cg=0.5−1	Light rockburst or spalling
Cg=1−2.5	High stress, slightly loosing
Cg>2.5	No rock stress induced instability

Table 15 shows the different advantages and limitations of this rockburst prediction criterion.

**Table 15:** Advantages and limitations present in the prediction method proposed by Palmström [112,113].

Advantage	Limitations
It evaluates the areas of weakness based on the size of that zone [114].	The value 1 is not clearly considered within a single classification range.
The estimation can be carried out using a set of input parameters [114].	It is based on the criterion of Hoek and Brown [5] for the classification of Cg, without considering that it is only applied to rock masses with an in-situ stress ratio of k = 0.5.
Quantitative form of predicting rockburst, thus reducing subjectivity and feasibility of qualitative assessment.	It does not consider the orientation factor of the discontinuities in the RMi or the Cg.
Field observations and measurements are useful for determining input parameters [114].	This system requires more calculations than other classification systems [114].
It considers the strength of the rock mass.	It estimates that rockburst only occurs in one type of competent and massive rock, without considering the probability of rockburst occurrence in blocky rocks
It considers the properties of discontinuities through the parameters of the RMi system.	Values of the competence factor may not always be representative due to rock anisotropy [112].
It considers the stresses present in the vicinity of the excavation.	It does not consider the impact of groundwater or rock stresses [114].
It evaluates by paying attention to the heterogeneity of the rock, that is, it allows analyzing the tunnel by advance or zone.	

### 5.8 Palmström [112]

The factor of competition, which characterizes failure modes in massive and brittle rocks, was developed based on stability evaluations conducted by Hoek and Brown [5], Russenes [67], and Grimstad and Barton [111]. Prior to considering these studies, a modification of the 1980 criterion was made to address the main stability issues observed in the walls of square tunnels in South Africa. In this regard, the author considered that the maximum tangential stress in the walls is approximately 1.4 $\sigma_{1}$, as pointed out by Hoek and Brown [5]. Therefore, the modification proposed by Palmström [112] is presented in Equation (12), along with its corresponding classification of rockburst activity in Table 16.

(12) $$\frac{\sigma_{\theta }}{\sigma_{c}}$$

where.

**Table 16:** Classification of rockburst levels after Palmström [112].

Criteria	Classification
$\frac{\sigma_{\theta }}{\sigma_{c}}<1.4$	Severe (sidewall) rockburst problems
$\frac{\sigma_{\theta }}{\sigma_{c}}=1.4$	Possible rockburst conditions
$\frac{\sigma_{\theta }}{\sigma_{c}}=1.7$	Heavy support required
$\frac{\sigma_{\theta }}{\sigma_{c}}=2.0$	Severe spalling
$\frac{\sigma_{\theta }}{\sigma_{c}}=3.5$	Minor (sidewall) spalling
$\frac{\sigma_{\theta }}{\sigma_{c}}>7$	Stable

$\sigma_{\theta }$ = Maximum tangential stress present around the tunnel

$\sigma_{c}$ = Uniaxial compressive strength of the rock

The table below shows the different advantages and limitations of the criterion modified by Palmström [112] (Table 17).

**Table 17:** Advantages and limitations present in the prediction method proposed by Palmström [112].

Advantage	Limitations
Simple mathematical equation.	It considered the uniaxial compressive strength of the rock, which is considered a standard strength that leads to very conservative analyses [95].
Easily obtainable input factors.	Does not mention the direction of the maximum tangential stress.
Quantitative form of predicting rockburst, thus reducing subjectivity and feasibility of qualitative assessment.	It considers the same real case considered by Hoek and Brown, without adding new observations that would allow for the proper updating of this criterion.
Quick calculation	It does not provide a range of values for each classification.
Classification developed based on the real case considered by Hoek and Brown [5].	It does not consider the properties of the discontinuities.
It modifies the criterion to consider the stresses that trigger stress-induced failure.	It does not consider the anisotropy or heterogeneity property of the rock.
	It still presents the same limitation of applying the Hoek and Brown criterion [5].
	When considering the uniaxial compressive Strength of the rock, the scale effect is overlooked, which considers the heterogeneity of the rock, such as microfractures and grains of different mineral compositions, when it comes to rock and rock mass strength. The existence of this scale effect on strength has been demonstrated by various author, such as [5,[103], [104], [105], [106], [107]].
	It estimates that rockburst only occurs in one type of competent and massive rock, without considering the probability of rockburst occurrence in blocky rocks.

### 5.9 Qiao and Tian [115]

The Brittle Index (Bi) is proposed based on an experimental study and in-situ research conducted in a copper mine in Dong-gua-shan, China. During the study, mechanical properties results were obtained for six different rock types, enabling the analysis of the probability and tendency of rockburst occurrence in the study area. Based on these findings, the authors propose a criterion and its corresponding classification (Table 18) based on Equation (13).

(13) $$\frac{\sigma_{c}}{\sigma_{t}}$$

where.

**Table 18:** Classification of rockburst levels after Qiao and Tian [115].

Criteria	Classification
$\frac{\sigma_{c}}{\sigma_{t}}<14.5$	Violent rockburst
$\frac{\sigma_{c}}{\sigma_{t}}=14.5-26.7$	Strong rockburst
$\frac{\sigma_{c}}{\sigma_{t}}=26.7-40$	Weak rockburst
$\frac{\sigma_{c}}{\sigma_{t}}>40$	No rockburst

$\sigma_{t}$ = Tensile Strength of the rock

$\sigma_{c}$ = Uniaxial compressive strength of the rock

This new criterion exhibits various advantages and limitations, which are presented in Table 19.

**Table 19:** Advantages and limitations present in the prediction method proposed by Qiao and Tian [115].

Advantage	Limitations
Simple mathematical equation.	It considered the uniaxial compressive strength of the rock, which is considered a standard strength that leads to very conservative analyses [95].
Easily obtainable input factors.	In the classification, the authors do not specify in which of the two ranges the value 26.7 falls.
Quantitative form of predicting rockburst, thus reducing subjectivity and feasibility of qualitative assessment.	It uses the tensile strength factor of the rock, which is of little importance because in-situ stresses at depth are never tensile but compressive [2,42].
Quick calculation.	It does not consider the properties of the discontinuities.
Classification developed based on the analysis of several real-life cases.	It does not consider the anisotropy or heterogeneity property of the rock.
	Presenting parameters that do not account for variations caused by underground excavation.
	When considering the uniaxial compressive Strength of the rock, the scale effect is overlooked, which considers the heterogeneity of the rock, such as microfractures and grains of different mineral compositions, when it comes to rock and rock mass strength. The existence of this scale effect on strength has been demonstrated by various author, such as [5,[103], [104], [105], [106], [107]].
	The criterion employs the concept of rock failure mode by tension (Mode I).

### 5.10 Martin et al. [108]

An analysis and study of the Hoek and Brown [5] criterion was conducted, and it was observed that their classification does not directly apply to other tunnel shapes that have a different in situ stress ratio (k) than 0.5. Additionally, the researchers noted that this criterion should not consider the major principal stress, but rather the maximum tangential stress present at the excavation boundary. This consideration is because the initiation of stress-induced failure begins in areas with high stress concentrations near the tunnel boundary.

To make this modification, it was necessary to estimate the effects of tunnel geometry and different stress ratios on the maximum tangential stresses. For this purpose, the solution proposed by Greenspan [116] was utilized, which allowed determining the maximum tangential stresses for the historical cases of Hoek and Brown [5]. The conversion of the authors' classification is presented in Table 20 in terms of Equation (14).

(14) $$\frac{\sigma_{\theta }}{\sigma_{c}}$$

where.

**Table 20:** Classification of rockburst levels after Martin et al. [108].

Criteria	Classification
$\frac{\sigma_{\theta }}{\sigma_{c}}\leq 0.4$	The rock mass is elastic and does not exhibit visible damage.
$\frac{\sigma_{\theta }}{\sigma_{c}}=0.65$	The rock mass is not elastic; therefore, stability is controlled by stress-induced damage in the roof. There is less spalling on the sidewall.
$\frac{\sigma_{\theta }}{\sigma_{c}}=0.95$	Severe spalling requiring moderate support.
$\frac{\sigma_{\theta }}{\sigma_{c}}=1.25$	Heavy support requirement for the opening to remain stable. Very severe spalling.
$\frac{\sigma_{\theta }}{\sigma_{c}}\geq 1.6$	Severe rockburst problems.

$\sigma_{\theta }$ = Maximum tangential stress present around the tunnel

$\sigma_{c}$ = Uniaxial compressive strength of the rock

In Table 21, some of the advantages and disadvantages of this criterion, which transforms the method proposed by Hoek and Brown [5], are presented.

**Table 21:** Advantages and limitations present in the prediction method proposed by Martin et al. [108].

Advantage	Limitations
Simple mathematical equation.	It considered the uniaxial compressive strength of the rock, which is considered a standard strength that leads to very conservative analyses [95].
Easily obtainable input factors.	Does not mention the direction of the maximum tangential stress.
Quantitative form of predicting rockburst, thus reducing subjectivity and feasibility of qualitative assessment.	It considers the same real case considered by Hoek and Brown [5], without adding new observations that would allow for the respective update of this criterion.
Quick calculation.	It does not provide a range of values for each classification.
Classification developed based on the real case considered by Hoek and Brown [5].	It does not consider the properties of the discontinuities.
It is applied for all in situ stress ratios (k), thus eliminating one of the most significant limitations of Hoek and Brown [5].	It does not consider the anisotropy or heterogeneity property of the rock.
It modifies the criterion to account for the stresses that trigger stress-induced failure.	It estimates that rockburst only occurs in one type of competent and massive rock, without considering the probability of rockburst occurrence in blocky rocks.
	When considering the uniaxial compressive Strength of the rock, the scale effect is overlooked, which considers the heterogeneity of the rock, such as microfractures and grains of different mineral compositions, when it comes to rock and rock mass strength. The existence of this scale effect on strength has been demonstrated by various author, such as [5,[103], [104], [105], [106], [107]].

### 5.11 Palmström [117]

This method presents the same prediction criterion as Palmström [112,113], but incorporates additional considerations for the application of the massiveness parameter ($f_{\sigma }$) in massive rocks. These considerations state that $f_{\sigma }\approx 0.5$ when Db>2m, approximately, while when $\text{JP}<f_{\sigma }$ (where JP<0.5 approx), the RMi equation is used for jointed rocks.

The proposed method uses the same classifications shown in Table 14 and highlights several advantages and limitations similar to those mentioned in Table 15.

### 5.12 Lee et al. [40]

It has been noted that displacements caused by high stresses in the rock, generated during underground construction, are comparable to the work done on the rock. If the rock exhibits elastic properties, these displacements can be stored as potential energy of deformation, with the possibility of being rapidly released in the form of rockbursts. In light of this, the authors consider that the occurrence of rockbursts depends on the ability of the rock mass to store elastic deformation energy. This ability can be observed in samples subjected to uniaxial compression and can be used to calculate the strain energy density, the strength index, and the energy index.

Based on this fundamental concept, they established a relationship between the strain energy density (developed by Kwasniewski and illustrated by Wang and Park [118]) and the fragility index (proposed by Qiao and Tian [115]). For this purpose, they used data obtained in the laboratory, literature review, and the study of the longest river tunnel in Korea, which was excavated using blasting (NATM) and a tunnel boring machine in three different types of rock. This correlation is expressed in equation (15), and the classification of strain energy density (SED) illustrated in Table 22 is used.

(15) $$\text{SED}=213.94\times \ln\left(B_{i}\right)-321.1$$

where.

**Table 22:** Classification of Strain Energy Density (SED) after Lee et al. [40].

Criteria	Classification
$\text{SED}=50\;\text{kJ}/m^{3}$	The rockburst hazard is very low
$\text{SED}=51\;∼100\;\text{kJ}/m^{3}$	The rockburst hazard is low
$\text{SED}=101\;∼150\;\text{kJ}/m^{3}$	The rockburst hazard is moderate
$\text{SED}=151\;∼200\;\text{kJ}/m^{3}$	The rockburst hazard is high
$\text{SED}>200\;\text{kJ}/m^{3}$	The rockburst hazard is very high

SED = Strain Energy Density ($\text{SED}=\frac{{\sigma_{c}}^{2}}{2\times E}$; where E is the tangential modulus of elasticity (MPa) and $\sigma_{c}$ is the uniaxial compressive strength of the rock (MPa))

$B_{i}$ = Fragility index

This criterion, which combines two different approaches, exhibits various advantages and limitations, which are presented in Table 23.

**Table 23:** Advantages and limitations present in the prediction method proposed by Lee et al. [40].

Advantage	Limitations
Simple mathematical equation.	The limitations of Qiao and Tian [115] are still present in this criterion.
Easily obtainable input factors.	It considers values from a minimum of $50\;\text{kJ}/m^{3}$.
Quantitative form of predicting rockburst, thus reducing subjectivity and feasibility of qualitative assessment.	Using the same SED classification without modifying it based on the new analyzed cases.
Quick calculation	It does not consider the properties of the discontinuities.
Relationship obtained from real cases of 12 rock types.	Evaluates and classifies assuming rock homogeneity and isotropy.
It shows a good correlation (coefficient of determination = 0.72).	
It considers the general concept of rockburst, that is, the energy.	

### 5.13 Bieniawski et al. [119]

A new criterion called Elastic Behavior Index (ICE) is presented, which allows predicting the behavior of tunnels constructed through drilling and blasting. This criterion is based on the equations proposed by Kirsch [99]. According to these equations, it is established that the ground is in the elastic domain when the following condition is met: $\frac{\sigma_{\text{cm}}}{\left(3-K\right)\times \gamma \times H}\;>1$ (with K≤1) o $\frac{\sigma_{\text{cm}}}{\left(3K-1\right)\times \gamma \times H}\;>1$ (with K≥1); where K is the coefficient relating in-situ stresses ($\sigma_{h}/\sigma_{v}$).

To evaluate and validate this criterion, the authors conducted an analysis of the ground behavior in four underground excavation cases. These cases were modeled using FLAC 3D software, which allowed them to classify the criterion based on the obtained results. The resulting classification is presented in Table 25 and is determined by applying Equation (16).

(16) $$\text{ICE}=100\times \frac{\sigma_{\text{cm}}}{\sigma_{\theta }}\times F$$

Where $\sigma_{\text{cm}}$, Uniaxial compressive strength of rock mass, adapted to the Kalamaras and Bieniawski [120] relationship, can be estimated from Equation 17

(17) $$\sigma_{\text{cm}}=\sigma_{c}\times e^{\frac{{\text{RMR}}_{89}-100}{24}}$$

Where $\sigma_{c}$ represents the uniaxial compressive strength of the rock and ${\text{RMR}}_{89}$ refers to the Rock Mass Rating by Bieniawski [121]

**Table 24:** Shape factor classification after Bieniawski et al. [119].

Underground excavation	F
*Circular tunnels with a diameter of 6 m*	1.3
*Circular tunnels with a diameter of 10 m*	1.0
*Conventional tunnels with a diameter of 14 m*	0.75
*Chamber with a width of 25 m and a height of 60 m*	0.55

**Table 25:** Classification of the Elastic Behavior Index after Bieniawski et al. [119].

Criteria	Classification
ICE<15	Mostly yielding
ICE=15−39	Intensive yielding
ICE=40−69	Moderate yielding
ICE=70−130	Elastic with incipient yielding
ICE>130	Completely elastic

The maximum tangential stress denoted as $\sigma_{\theta }$ is derived using Kirsch's equations [99]. This stress value is determined in relation to a baseline value of K, which is calculated based on Equations (18), (19)).

Where $\sigma_{v}$ the vertical principal stress

(18) $$\text{For}\;K\leq 1\;\sigma_{\theta }=\left(3-K\right)\times \sigma_{v}$$

(19) $$\text{For}\;K\geq 1\;\sigma_{\theta }=\left(3K-1\right)\times \sigma_{v}$$

F = Shape factor that can take on the values from Table 24. It is considered to allow the application of the Kirsch equations to various tunnel shapes, not just circular excavations.

This new ICE criterion presents various advantages and limitations, as mentioned in Table 26.

**Table 26:** Advantages and limitations present in the prediction method proposed by Bieniawski et al. [119].

Advantage	Limitations
It can assess the behavior of various forms of underground excavation.	They use a system that does not accurately estimate the quality of rock masses exhibiting swelling, squeezing, or popping ground behavior [98].
Easily obtainable input factors.	It only provides four values of the shape factor.
Quantitative form of predicting rockburst, thus reducing subjectivity and feasibility of qualitative assessment.	The values of the parameter F are only applicable to civil constructions, not to mining.
It evaluates by paying attention to each advance or zone of the tunnel, meaning it presents parameters that assess to some extent the heterogeneity of the rock.	As the RMR_89_ system does not reflect the true nature of the rock mass [122], neither will the ICE when considering it as one of the input parameters.
It considers the strength of the rock mass.	The classification system does not consider the anisotropy or heterogeneity of the rock [100,101].
Simple mathematical equation.	They classify the ICE values based on the results of numerical modeling, without considering investigations of the behavior of real underground excavations.
It considers the properties of the discontinuities.	It does not have a physical meaning for in situ stress ratios k < 2.
Quick calculation.	It considers the uniaxial compressive strength of the rock in the RMR system, which is considered as a standard strength leading to highly conservative analyses [95].
It allows evaluating the prediction in both poor-quality and good-quality rock masses since RMR_89_ is effective in estimating the quality in this type of rock masses [123].	

### 5.14 Criteria of barton et al. [38], russenes [67] and Hoek and Brown [5] modified by zhao et al. [61]

It has been observed that the various criteria used to predict and classify the intensity of rockburst can yield inconsistent results compared to actual field events. For this reason, it is proposed to modify the three traditional methods using 29 documented cases of rockburst observations in the Qirehataer diversion tunnel as a basis. The main objective is to improve the consistency of the classifications by verifying the relationships between the principal stresses and the strength, as described in the different criteria.

To achieve this, the authors make specific changes to each criterion based on the findings of Zhao et al. [61]. These changes are presented in detail in Table 27, while the advantages and limitations associated with the modification of these criteria are listed in Table 28. It is important to note that the authors consider that the parameters $\sigma_{v}$ and $\sigma_{\theta }$ have the same meaning when considering the modification proposed by Palmström [112], which affects the original criterion of Hoek and Brown [5] that uses $\sigma_{1}$ and $\sigma_{h}$.Where.

**Table 27:** Classification and modifications of rockburst levels after Zhao et al. [61].

Author	Criteria	Classification
Barton et al. [38]	$\frac{\sigma_{c}}{\sigma_{1}}\leq 1.5$	No rockburst
	$1.5<\frac{\sigma_{c}}{\sigma_{1}}\leq 2.5$	Light rockburst
	$2.5<\frac{\sigma_{c}}{\sigma_{1}}\leq 4$	Moderate rockburst
	$4<\frac{\sigma_{c}}{\sigma_{1}}\leq 5$	Strong rockburst
	$\frac{\sigma_{c}}{\sigma_{1}}>5$	Very strong rockburst
Russenes [67]	$\frac{\sigma_{\theta }}{\sigma_{c}}\leq 0.2$	No rockburst
	$0.2<\frac{\sigma_{\theta }}{\sigma_{c}}\leq 0.5$	Light rockburst
	$0.5<\frac{\sigma_{\theta }}{\sigma_{c}}\leq 0.7$	Moderate rockburst
	$0.7<\frac{\sigma_{\theta }}{\sigma_{c}}\leq 0.9$	Strong rockburst
	$\frac{\sigma_{\theta }}{\sigma_{c}}>0.9$	Very strong rockburst
Hoek and Brown [5]	$\frac{\sigma_{c}}{\sigma_{v}}\leq 2.5$	No rockburst
	$2.5<\frac{\sigma_{c}}{\sigma_{v}}\leq 3.3$	Light rockburst
	$3.3<\frac{\sigma_{c}}{\sigma_{v}}\leq 5$	Moderate rockburst
	$5<\frac{\sigma_{c}}{\sigma_{v}}\leq 10$	Strong rockburst
	$\frac{\sigma_{c}}{\sigma_{v}}>10$	Very strong rockburst

**Table 28:** Advantages and limitations present in the prediction method proposed by Zhao et al. [61].

Advantage	Limitations
Simple mathematical equation.	It considered the uniaxial compressive strength of the rock, which is considered a standard strength that leads to very conservative analyses [95].
Easily obtainable input factors.	Applied only for a stress ratio greater than 1.
Quantitative form of predicting rockburst, thus reducing subjectivity and feasibility of qualitative assessment.	It applies the maximum in-situ stress, despite being unreasonable to apply in prediction criteria, as according to Kirsch [99], $\sigma_{\theta }$ progressively decreases from $3\sigma_{1}-\sigma_{3}$ to $\sigma_{1}$ with distance from the tunnel. Additionally, the resulting ${\sigma_{\theta }}^{\max}$ is influenced by excavation-induced stresses [65].
Quick calculation.	There is inconsistency in classification when using the three criteria in the same study area.
Classification developed based on the analysis of multiple real-life cases.	It does not consider the properties of the discontinuities.
Consistency observed between the results obtained from the criteria and the field observations.	It does not consider the anisotropy or heterogeneity property of the rock.
It presents a clear range of values in the classification of the Hoek and Brown criterion [5].	The modifications may not have a relationship with the lithology of the studied rock mass. This is caused by considering only one type of lithology in the base case.
It is verified that the changes made to the criteria improved the coherence of the criteria.	Rockburst is defined solely by the mechanical properties of the rock.
	It can only be applied in a reverse stress regime.
	When considering the uniaxial compressive Strength of the rock, the scale effect is overlooked, which considers the heterogeneity of the rock, such as microfractures and grains of different mineral compositions, when it comes to rock and rock mass strength. The existence of this scale effect on strength has been demonstrated by various author, such as [5,[103], [104], [105], [106], [107]].
	It considers that rockburst occurs in rocks under high stresses, which are brittle, massive, and located at great depths, but it does not assess the probability of rockburst occurrence in blocky rocks.
	Considering that $\sigma_{v}$ and $\sigma_{\theta }$ have the same meaning.

$\sigma_{\theta }$ = Maximum tangential stress or in situ vertical stress

$\sigma_{c}$ = Uniaxial compressive strength of the rock

$\sigma_{v}$ = In situ vertical stress or major principal stress. The authors considered that this parameter has the same definition as the major horizontal principal stress

### 5.15 Qiu and Feng [93]

Reference was made to the research by Qiu et al. [124], which presents a new criterion for assessing vulnerability to rockburst in deep excavations, known as the Rockburst Vulnerability Index (RVI). This method is based on four key uncoupled factors that estimate the behavior of the underground excavation, as shown in equation (20). Increasing RVI values indicate a higher impact of these factors on increasing the predisposition to rockburst. Furthermore, the score assigned to each factor determines the relative contribution and the mathematical relationship by which RVI values increase as the tendency for rockburst increases.

(20) $$\text{RVI}=F_{s}F_{r}F_{m}F_{g}$$

where.

$F_{s}$ = Stress control factor is a parameter that considers the in-situ stress conditions in the rock and is crucial for controlling and assessing the possibility of rockburst (equation (21)). Using vertical stress ($\sigma_{v}$) or maximum principal stress ($\sigma_{1}$) alone may overlook the shape and size effects of the underground excavation, leading to an overestimation of the failure probability. Therefore, it is appropriate to utilize the maximum tangential stress ($\sigma_{\theta }$) obtained through numerical methods, which prevents the direct application of $\sigma_{\theta }$ in existing empirical indices. To this end, the authors propose a new numerical method that evaluates the effect of stresses on rockburst, as shown in the following formula that determines the stress condition in the vertical plane to the tunnel axis.

(21) $$F_{s}=\frac{100\;\sigma_{v}}{\sigma_{c}}\left(\text{Ak}+B\right)\;\left(A=f_{1}\left(\beta \right);\;B=f_{2}\left(\beta \right)\right)$$

The symbols represent.

k = Relationship between the horizontal stress and the vertical stress present in the in-situ stress field of the rock.

$\sigma_{v}$ = Vertical stress

$\sigma_{c}$ = Uniaxial compressive strength of the rock

AyB = Shape and size coefficients of underground excavations, where $f_{1}$ and $f_{2}$ will indicate these functions, respectively

$F_{r}$ = Petrophysical factor. A more detailed assessment of rockburst vulnerability is conducted compared to the brittleness index. This assessment is carried out from the perspective of petrophysical properties and levels of damage resistance caused by high stresses in rocks. It describes the impact of rock's mechanical properties, considering the heterogeneity present in mineral compositions and different particle sizes. This factor is mathematically expressed as shown in Equation (22).

(22) $$F_{r}=M_{h}\;G_{s}$$

The symbols represent.

$M_{h}$ = Mineral heterogeneity factor (Table 29), determined from the stiffness or strength heterogeneity factor (SHF) or the characteristics of minerals present in the rock under study.

**Table 29:** Heterogeneity factors score $M_{h}$ after Qiu and Feng [93].

Characteristics of Jinping marbles	*Score of parameter*$M_{h}$	
	*Factor SHF*	$M_{h}$*Score*
*Grayish-white fine-grained marble*	1.39	1.0
*Grayish-white marble with medium-coarse grains*	1.35	1.2
*Grayish-black and white fine-grained marble*	1.30	2.0
*Grayish-black and white marble with medium-coarse grains*	1.34	1.6

$G_{s}$ = Grain size or particle size factor of the rocks (Table 30).

**Table 30:** Grain size score $G_{s}$ after Qiu and Feng [93].

Characteristics of Jinping marbles	*Score of parameter*$G_{s}$		
	*Grain size (mm)*	$G_{s}$*Score*	*Description*
*Grayish-white fine-grained marble*	<0.1	1.0	Micrograin
*Grayish-white marble with medium-coarse grains*	0.1–1.0	1.2	Fines grain
*Grayish-black and white fine-grained marble*	1.0–3.0	1.4	Medium grain
*Grayish-black and white marble with medium-coarse grains*	>3.0	1.4–2.0	Coarse grain

$F_{m}$ = Stiffness of the system factor (Table 31). It is based on the concept of Local Mine Stiffness (LMS) proposed by Kaiser et al. [125], which evaluates the stiffness of the system that connects the areas to be excavated with the adjacent areas that have already been disturbed due to underground construction. This factor describes the ability of stresses to release or respond to deformations present in the areas to be excavated, considering the design of the engineering construction, implications of the geometry of the excavation zone, and stress redistribution capacity.

**Table 31:** Score to parameter $F_{m}$ after Qiu and Feng [93].

*Parameter score*$F_{m}$	Description of excavation environment
1.0	Excavation environment with high stiffness: Single planes of undisturbed rock are excavated or the areas to be excavated are two times of the tunnel diameter to excavated structures. In such condition, the excavation is slightly affected by excavated structures
1.5	Excavation environment with medium stiffness: areas to be excavated are located in the adjacent to excavated structured and stress in the areas are relieved in at least one direction
2.0	Excavation environment with low stiffness: the areas influenced by two or more excavated structures (stress concentration areas for example) are excavated, and the areas release stress and are deformed in multiple directions.

$F_{g}$ = Geological structure factor (Table 32). It considers regional and local structures that control the occurrence of rockburst. These structures have significant impacts on rockburst, which are reflected in two aspects.

a. In the cores of syncline folds and in the two limbs of anticline folds, higher in situ stresses are observed compared to other fold conditions. These folding structures can also alter the direction of the stress field and the relationship between the maximum and minimum principal stresses. Additionally, in the study areas, other types of geological structures can be found, such as mechanical discontinuity planes. These planes can accumulate a significant amount of deformation energy, especially between the discontinuity surface and the excavation faces.
b. Geological structures cause sliding and energy release on rigid structural surfaces. Shear-type sliding occurs as a result of fault activity or compressive structural surfaces. The activation of these types of structures can occur in two ways. The first is by decreasing the normal pressure on the structural surfaces, while the second occurs through an increase in the driving tangential force on these structural surfaces.

**Table 32:** Score for the parameter $F_{g}$ after Qiu and Feng [93].

*Parameter score*$F_{g}$	Description of geological structure characteristics
1.0	Excavation environment with high stiffness: Single planes of undisturbed rock are excavated or the areas to be excavated are two times of the tunnel diameter to excavated structures. In such condition, the excavation is slightly affected by excavated structures
2.0	Excavation environment with medium stiffness: areas to be excavated are located in the adjacent to excavated structured and stress in the areas are relieved in at least one direction
3.0	Excavation environment with low stiffness: the areas influenced by two or more excavated structures (stress concentration areas for example) are excavated, and the areas release stress and are deformed in multiple directions
4.0/5.0	Rock masses in the areas to be excavated are intact, and working faces are near to faults (possibly inducing fault dislocation) and influencing the areas of structures, such as faults. Moreover, there are small quantities of stiff structural surfaces with poor extension, including joints and fracture surfaces, or excavation surfaces are close to the tips of weak structural surfaces (twist-compressional joints and fracture surfaces)^a^

a Note: When working faces are close to the fault areas, $F_{g}$ was scored as 4.0; when the working faces approach the structural areas of folds, $F_{g}$ was valued as 5.0. The term “approach” refers to the distance within two times of the excavated tunnel diameter.

This multifactor criterion has various advantages and limitations, which are mentioned in Table 33.

**Table 33:** Advantages and limitations present in the prediction method proposed by Qiu and Feng [93].

Advantage	Limitations
Simple mathematical equation.	There is a degree of subjectivity in the parameters $F_{g}$ and $F_{m}$ when characterizing the rock based on field observations.
Easily obtainable input factors.	Recommended for experienced geologists and engineers.
Quantitative form of predicting rockburst, thus reducing subjectivity and feasibility of qualitative assessment.	It only considers the main behaviors of the excavation and geological structures.
Quick calculation.	It illustrates the characteristics of a single rock type, in this case, marble.
It is created based on the main parameters that affect the behavior of the rock mass.	It does not present the properties of the discontinuities.
It evaluates by paying attention to the heterogeneity of the rock.	It evaluates and classifies assuming rock isotropy.
It considers the damage caused by underground construction.	The estimation of rock mass strength is missing.
It is classified through the study of a real excavation.	
It allows evaluating the probability of rockburst in excavations of various shapes and sizes.	

#### 5.15.1 Kaiser [126], Kaiser and Moss [127]

**Fig. 7:**
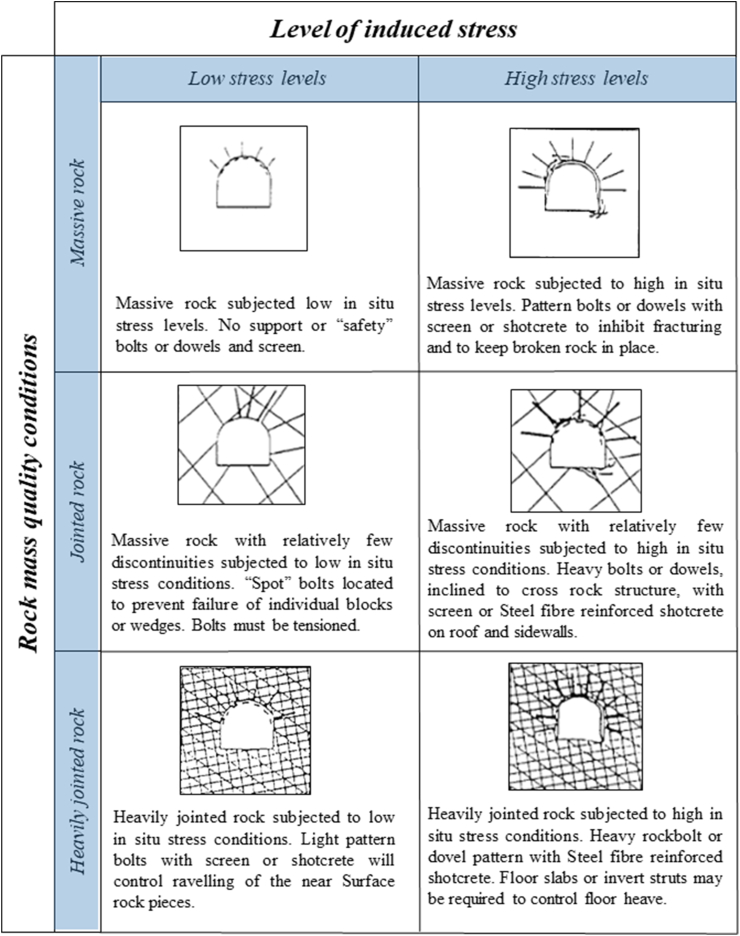
Excavation behavior matrix (modified after Kaiser and McCreath (1994)).

Kaiser and McCreath [6] support the claim that the factors considered by Hoek et al. [128] allow predicting the behavior of a rock mass in underground excavations. These parameters identify six basic behaviors of the rock mass, classified according to the properties of the mass in response to the levels of stress acting on it, as shown in the matrix in Fig. 7. The authors mentioned that the quality of the rock mass can be evaluated using the Rock Mass Rating (RMR) system, Tunneling Quality Index (Q), or Rock Quality Designation (RQD). Therefore, the behavior of the three types of rock quality under moderate stresses was evaluated. This study proposed that the behaviors can be described, from top to bottom, as exceptional to very good quality, good to fair quality, and poor to extremely poor quality.

**Fig. 8:**
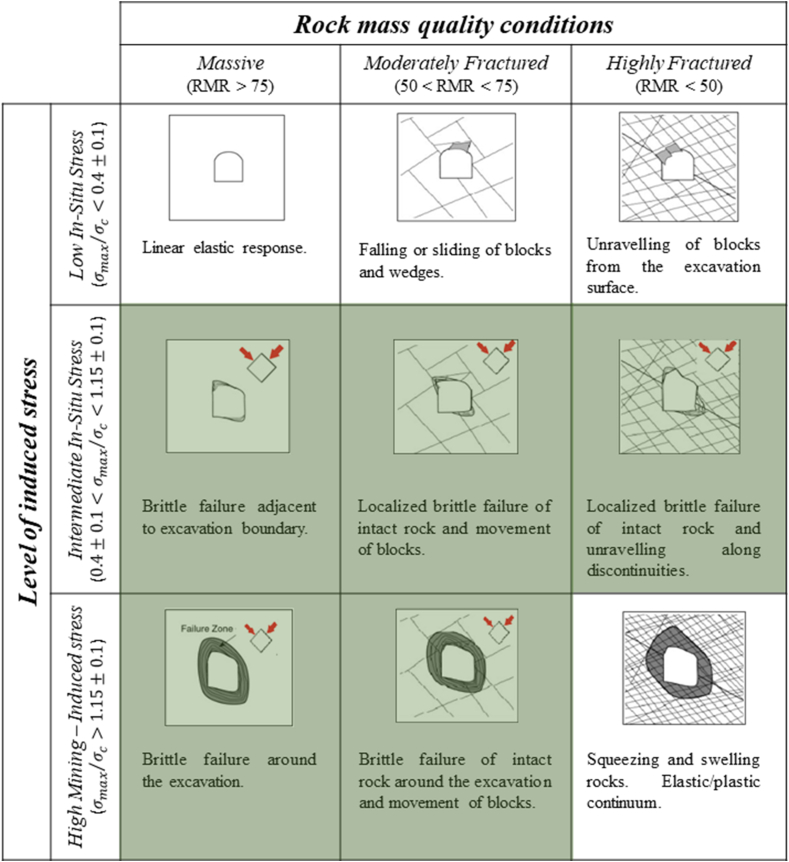
Excavation behavior matrix (modified after Kaiser (2000)).

Kaiser et al. [2] updated the behavior matrix presented by Kaiser and McCreath [6]. This new matrix includes nine types of instability modes in hard rock underground excavations, classified according to the stress level proposed by Martin et al. [108] and the rock mass quality defined by the RMR system, as shown in Fig. 8. The matrix highlights the behavior modes associated with brittle failure using gray-colored boxes, while the shear failure modes are the missing ones.

**Fig. 9:**
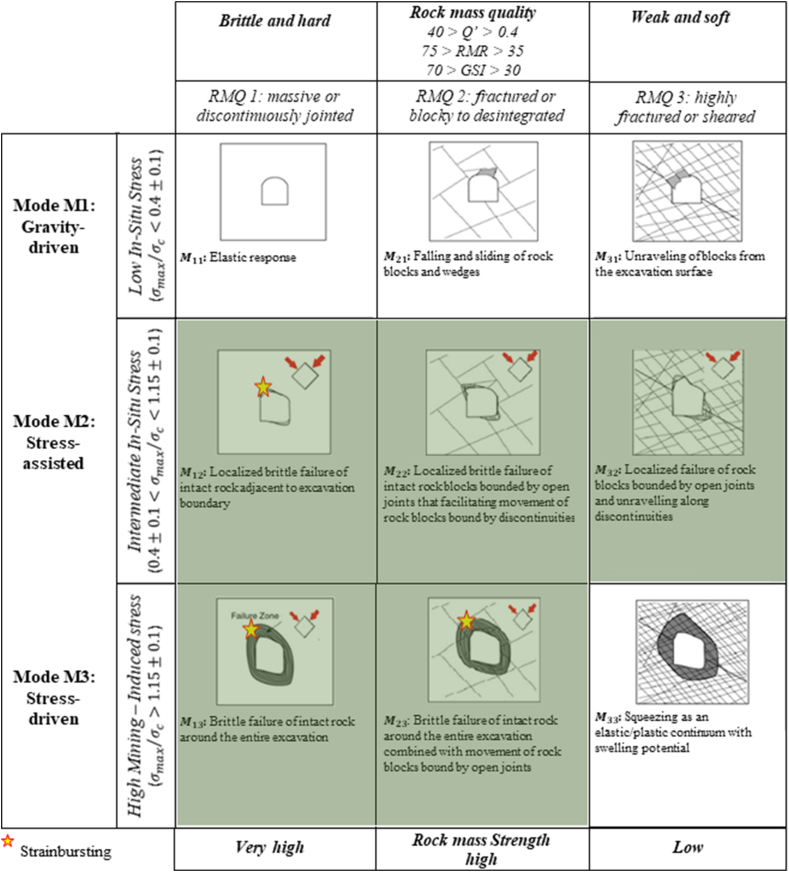
Excavation behavior matrix (modified after Kaiser (2018)).

Later, Kaiser [126] points out that the matrix presented by Kaiser et al. [2] describes the behaviors observed under static loading conditions. However, the author modifies this matrix by incorporating additional descriptions in each instability mode, based on the quality or strength of the rock mass and the stress level, as shown in Fig. 9. By investigating the three vertical classifications, according to the stresses present in the ground, it was found that:

- M_31_, M_32_ and M_33_: These are excavations constructed in highly fractured or sheared ground, which are prone to falls of ground and excessive plastic deformations. They are characterized by having limited unsupported strength. Furthermore, rock masses subjected to high stress levels (M_32_ y M_33_) will require a robust support system.
- M_21_, M_22_ and M_23_: These are underground constructions developed in fractured or blocky-to-disintegrated rocks, which are prone to failures with structural control. In low-stress zones (M_21_), support systems will be required, especially when the roof relaxes due to the low stress ratio k ($\sigma_{h}/\sigma_{v}$). However, at high stress levels (M_22_ and M_23_), blocks formed by open joint sets will fracture near the excavation due to the extension of deformation caused by stress heterogeneity.
- M_31_, M_32_ and M_33_: Excavations developed in massive to discontinuous jointed terrains, prone to fracturing due to stress concentration near the excavation. At shallow depths, high-quality rocks respond elastically. However, at high stress levels, they may exhibit localized spalling or stress-induced fracturing, leading to the formation of notches.

**Fig. 10:**
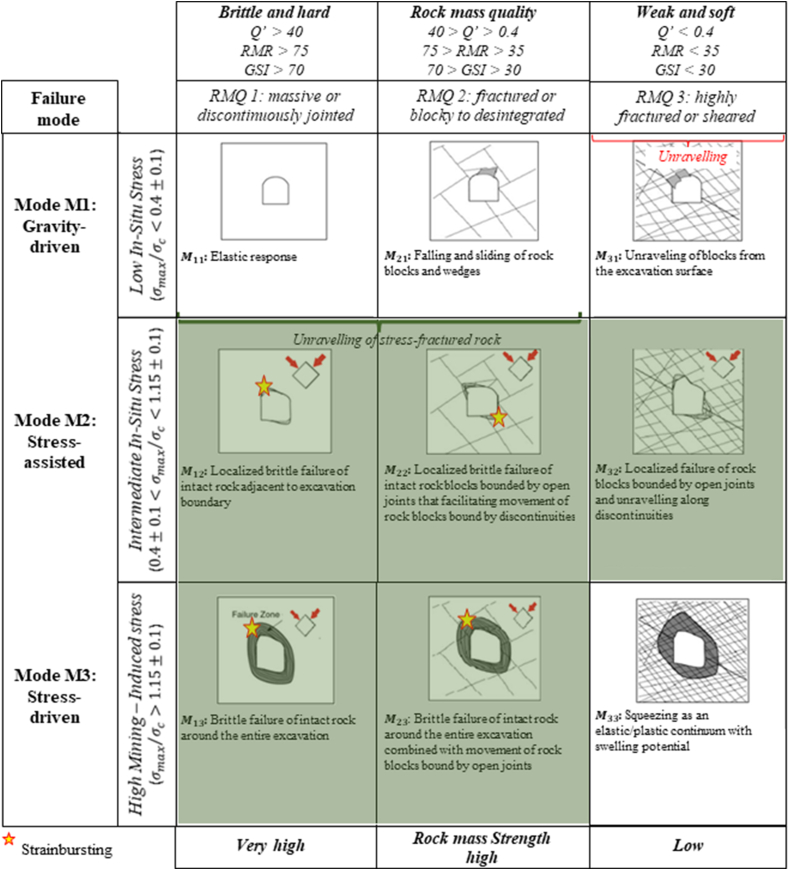
Excavation behavior matrix (modified after Kaiser and Moss (2022)).

Kaiser and Moss [127] made modifications to instability mode M_22_ of the matrix proposed by Kaiser [126]. In their study, they indicated that this behavior would be associated with a strainburst-type rockburst, as illustrated in Fig. 10.

This criterion presents various advantages and limitations of application, as presented in Table 34.

**Table 34:** Advantages and limitations present in the prediction method proposed by Kaiser [126] and Kaiser and Moss [127].

Advantage	Limitations
Simple mathematical equation.	They use the RMR system, which does not accurately estimate the quality of rock masses exhibiting swelling, squeezing, or popping ground behavior [98].
Easily obtainable input factors.	It is not mentioned whether the quality of the rock mass is obtained from a quantitative or qualitative GSI system.
Quantitative form of predicting rockburst, thus reducing subjectivity and feasibility of qualitative assessment.	The parameters of Q’ (J_r_, J_a_, J_n_ and RQD) are subjective as they are obtained from observations [96].
Quick calculation.	It evaluates and classifies the prediction of rockburst assuming isotropy and homogeneity of the rock, as the existing systems do not consider it in estimating the rock mass quality [[100], [101], [102]].
It considers the strength of the rock mass.	Based on research that does not consider all the key parameters to predict the mode of instability of the ground.
Allows for the analysis of tunneling advancement.	The RQD/J_n_ and J_w_/SRF ratios of the Q′ system do not provide a meaningful measure of block size and the acting stresses in rock masses, respectively [96,129].
It considers the properties of the discontinuities.	They make it difficult to correlate RQD with other parameters that measure jointing when evaluating the rock mass in a one-dimensional manner, specifically within a core sample of no more than 0.1 m [130].
It is created based on the main parameters that affect the behavior of the rock mass.	It considers that rockburst occurs in rocks under high stresses, that are brittle, massive, and located at great depths, but it does not evaluate the probability of rockburst occurrence in blocky rocks.
	By using the matrix presented by Kaiser and McCreath [6] as the basis for updating, it assumes three generic categories. of typical ground conditions that occur during hard rock drilling.
	It considered the uniaxial compressive strength of the rock, which is considered a standard strength that leads to very conservative analyses [95].
	When considering the uniaxial compressive Strength of the rock, the scale effect is overlooked, which considers the heterogeneity of the rock, such as microfractures and grains of different mineral compositions, when it comes to rock and rock mass strength. The existence of this scale effect on strength has been demonstrated by various author, such as [5,[103], [104], [105], [106], [107]].

Based on the above detail literature review about the prediction of rock burst, we can brief our observations as following: among all the approaches 10 of them are based on the theory of rock strength. Strength theory, a traditional criterion for material failure, posits that failure in a material commences when the stress imposed surpasses its strength threshold. All those criteria are typically described in terms of the ratio between the state of stress represented by maximum in-situ stress or induced hoop tress and the strength of the rock which consider by the factors UCS or Ut of the rock. Nevertheless, many of these strength criteria overlook the intermediate principal stress, which plays a crucial role in determining the strength at which rock failure occurs. However, in each approach the value of this ratio is different from another. These methods considers the strength of the intact rock at laboratory scales and there is no procedure to up scale into tunnel applications.

6. Case study

The New Level Mine Project (PNNM) at El Teniente is a sophisticated underground mining endeavor designed to extract reserves located beneath the Teniente Sub-6 level (elevation 2101 m) and the Esmeralda level (elevation 2210 m), while the Sinking Level is situated at an elevation of 1880 m. Over the years, the mine has faced numerous rock bursting incidents. These incidents have varied in severity, ranging from minor disruptions to substantial bursts that inflict extensive damage on the mine's infrastructure and present significant hazards to the mining workforce. The occurrence of rock bursts in this mine is influenced by several factors, including the depth of mining operations, geological conditions such as fault presence and rock type, as well as the methodologies employed in mining.

To counteract the risks associated with rock bursts, El Teniente has implemented a range of monitoring and prediction techniques. This includes the use of seismic monitoring systems to identify the minor tremors that frequently signal an impending rock burst, along with stress measurement and modeling efforts aimed at comprehensively understanding the stress distribution within the rock mass. Additionally, the mine utilizes hydraulic fracturing techniques as a proactive measure to reduce the likelihood of rock bursts.

El Teniente's experience with rock bursting highlights the challenges faced in deep underground mining and the importance of advanced engineering, monitoring, and safety practices to protect miners and infrastructure. The continual development of more sophisticated prediction and mitigation techniques is crucial for enhancing safety in such extreme mining conditions. The pivotal role of ongoing research and technological advancement cannot be overstated in enhancing the effectiveness of rock burst prediction and mitigation strategies at early stage of the development with few available geotechnical data set. In this vein, El Teniente actively collaborates with various academic and research institutions, leveraging their expertise to improve safety and operational efficiency in facing these geotechnical challenges.

**Fig. 11:**
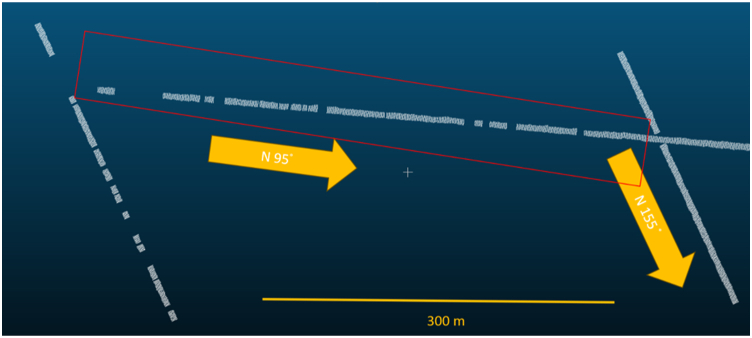
The study area in the Sinking Level of the New Level Mine Project.

The place under study is located 100 m below the deepest level of the mine (Teniente 8). The mining method employed is Panel Caving with Advanced Sublevel Caving in primary rock. The Sinking Level is located at an elevation of 1880, while the Transport Level, connecting NNM with the Colón Plant, is at an elevation of 1713. Additionally, a high degree of mechanization is implemented at all levels of this project. The present study was conducted in the Sinking Level (UCL) of the New Level Mine Project, specifically in the section delineated by dashed red lines (Fig. 11). This section comprises three subsections, where the parallel sections, with an orientation of N 95° E, are connected by a third subsection, oriented N 155° E.

Table 35 displays the measurements of in situ stress at various tunnel locations, whereas Table 36 outlines the geomechanical characteristics of the rock mass determined through laboratory tests. Taking into account the in-situ stress, tunnel direction, and tunnel diameter, the maximum value of the hoop stress around the tunnel perimeter is estimated using Kirch equations as a crucial aspect of this study. According to Kirch equation, the maximum stress around the tunnel occurs at an angle of 82°, with the corresponding uniaxial strength of the rock mass measuring 121.39 MPa. Conversely, the minimum stress is obtained at an angle of 352°, with the corresponding uniaxial strength of the rock mass being 46.42 MPa.

**Table 35:** Measurements of in situ stress at various tunnel locations.

Sxx	Syy	Szz	Sxy	Sxz	Syz
51.2	51.8	30.7	−6.5	3.1	−0.5
51.5	52.0	31.0	−6.8	3.2	−0.6
51.7	52.3	31.0	−7.0	3.2	−0.8

**Table 36:** Parameter geotechnical of the New Level of El Teniente Mine, Chile.

GEOTECHNICAL PROPERTIES OF EL TENIENTE MINE	
*Parameters*	*Value*
Uniaxial compressive strength of the rock	120 MPa
Tensile Strength of the rock	14 MPa
Maximum tangential stress present around the tunnel	121 MPa
Minimum principal stress in-situ	30.3 MPa
Maximum principal stress in-situ	59.2 MPa
Span	4 m

## 6 Application of reviewed methods to predict rock bursts and their results

The aforementioned approaches for predicting rock bursts were applied to 54 tunnel sections at El Teniente mine. However, when these criteria were applied to the specific case of the New level of El Teniente mine in Chile, a notable disparity was observed in the estimated occurrences of rock bursts and their severity. These results are presented in Table 37, which shows that only five of the approaches allow prediction by advancement, meaning they consider certain parameters to some extent that account for rock heterogeneity. On the other hand, the other approaches predict that the rockburst intensity will be the same and occur throughout the underground excavation.

**Table 37:** Rockburst predictions obtained when applying the studied empirical criteria in the case study.

Author	Value	Classification
Barton et al. [38]	$\sigma_{c}/\sigma_{1}=2.027$	Heavy rockburst
	$\sigma_{t}/\sigma_{1}=0.236$	Mild rockburst (massive rock)
Russenes [67]	1.008	Strong rockburst
Hoek and Brown [5]	0.493	Possible rockburst condition or heavy support required
Turchaninov et al. [109]	1.502	Violent rockburst
Zhen-Yu [110]	2.027	Strong rockburst
Grimstad and Barton [111]	$\sigma_{c}/\sigma_{1}=2.027$	It can result in slabbing and rockburst after minutes in massive rock or heavy rockburst (strainburst) and immediate dynamic deformations in massive rock
	$\sigma_{\theta }/\sigma_{c}=1.008$	
Palmström [112,113]	–	88% of the continuous advancements exhibit light rockburst or spalling
		12% of the continuous advancements exhibit heavy rockburst
Palmström [112]	0.992	Severe (sidewall) rockburst problems
Qiao and Tian [115]	8.571	Violent rockburst
Martin et al. [108]	1.008	Severe spalling in the opening or heavy support required to stabilize the opening. Brittle fracture initiation when greater than 0.4
Palmström [117]	–	68% of the continuous advances exhibit light rockburst or spalling
		32% of the continuous advances exhibit heavy rockburst
Lee et al. [40]	138.536	The rockburst hazard is moderate
Bieniawski et al. [119]	–	81.5% of the advances will exhibit stress-deformation behavior of intensive yielding, while 18.5% will mostly yield, considering the limitation of RMR_89._
		96.3% of the advances will exhibit stress-deformation behavior of intensive yielding, while 3.7% will show moderate yielding, without considering the limitation of RMR_89._
Criteria of Barton et al. [38] modified by Zhao et al. [61]	2.027	Strong rockburst
Criteria of Russenes [67] modified by Zhao et al. [61]	1.008	Very strong rockburst
Criteria of Hoek and Brown [5] modified by Zhao et al. [61]	2.027	Very strong rockburst
Qiu and Feng [93]	291.336	74.07% of the advances obtain this RVI value
	437.004	25.93% of the advances obtain this RVI value
Kaiser [126], Kaiser and Moss [127]	–	*Global estimation:* It may exhibit strainburst and localized brittle failure of intact rock blocks bounded by open joints that facilitate rock movement along the discontinuities (M_22_)
		The local estimation obtained by considering Cai and Kaiser [131] evaluated that 100% of the advances would exhibit M_22_ behavior mode
		The local estimation using the quantitative GSI method proposed by Russo [132] evaluated that 25.93% of the advances could exhibit an M_21_ mode, 66.67% showed an M_22_ behavior, and 7.41% would display and M_21_ or M_22_ mode as they lie on the boundary between both behaviors
		*The local estimation using the RMR system* predicts that 83.33% of the sequences will exhibit M_22,_ mode behavior, 3.7% will exhibit M_21_ mode behavior, and 12.96% may exhibit either M_21_ or M_22_ mode behavior
		*The local estimation obtained from Q′* evaluates that 14.81% of the advancements will exhibit M_21_ mode behavior, and 85.19% will exhibit M_22_ mode behavior

Barton et al. [38] proposed a prediction method that can be evaluated in two ways: by using the relationship between the uniaxial compressive strength of the rock and the maximum in situ principal stress, or the relationship between the tensile strength of the rock and the maximum in situ principal stress. When applying both criteria, it is observed that they estimate different intensities of rockburst, with the former predicting a higher intensity in the study area. This may align with the observations of Grimstad and Barton [111], who noted an inconsistency in assigning a score to the SRF factor used in the calculation of the tunnel quality index (Q). This inconsistency arises from the fact that hard and massive rocks under high stresses require a higher level of support than what is recommended by the corresponding Q value.

The estimation obtained using the criterion proposed by Hoek and Brown [5] predicts that the study area may have a possibility of rockburst, or heavy support required. This evaluation arises from the possibility that the result falls within both classification ranges, considering only one value for each of the predictions in Table 6. This method can generate uncertainty for geomechanical engineers attempting to predict rockburst in an underground excavation based on this empirical criterion.

When applying the modification of the parameters used for the classification of the SRF factor, proposed by Grimstad and Barton [111], it is observed that both empirical methods predict rockburst in the same manner. Despite this, the result presents a degree of uncertainty and prediction certainty, as it falls between two classification ranges that estimate the possible occurrence of slabbing and rockburst after minutes in massive rock or heavy rockburst (strainburst) and immediate dynamic deformations in massive rock.

The criterion proposed by Palmström [112,113] and Palmström [117] has fewer limitations than the other empirical methods and is the only one that estimates the occurrence of rockburst in only 75.93% of the advancements. These methods differ only in the methodology used to obtain the Rock Mass Index (RMi), specifically in the application of the massiveness parameter, which leads to a difference in the percentages of advancement classified or intensity estimated.

Martin et al. [108] modifies the criterion proposed by Hoek and Brown [5], but by not presenting a range of values for each classification, but rather a single value, many results fall between two possible evaluations of rockburst, as observed in the studied tunnel where the possible occurrence of spalling in the opening or heavy support required to stabilize the opening is estimated.

The application of the criterion by Bieniawski et al. [119] uses the RMR system by Bieniawski [121] as an input parameter to estimate the rock mass strength according to the equation proposed by Kalamaras and Bieniawski [120]. In doing so, two situations were considered when calculating the rock mass quality: one considering that the ratings for the discontinuity spacing parameter must have ≥3 sets of discontinuities to be applied [121], and another that does not have this limitation on the number of sets for this input parameter. This is based on research conducted by Laubscher [133], which demonstrated that rock masses with fewer than three sets of discontinuities will be estimated conservatively by increasing the spacing parameter by 30%.

The rockburst vulnerability index allows evaluating the behavior of each excavation advance. However, it was estimated that the values of $F_{s}$, $F_{r}$ and $F_{g}$ are constant throughout the tunnel, and $F_{m}$ has only two possible stiffness values that vary in certain excavation advances. This resulted in the criterion estimating only two possible behaviors along the entire length of the tunnel.

Kaiser [126], Kaiser and Moss [127] are the only authors who consider more than one classification system to characterize the rock mass quality, which are RMR, Q′, and GSI. For this research, the following classification systems proposed by the following authors were applied:

- RMR = Proposed by Lowson and Bieniawski [134]. In the research, the system presented by Celada et al. [135] was not used, as it would lead to a double consideration of stress levels.
- Q’ = Grimstad and Barton [111].
- GSI = Quantitative systems of the Geological Strength Index proposed by Cai and Kaiser [131] that consider block volume as an input parameter in their formulation, and Russo [132] that considers the discontinuity parameter of the Rock Mass Index (RMi) [113]. It is important to note that the block volume was obtained from the discrete fracture network (DFN) model.

In the research, it was decided to estimate the prediction of rockburst at both global and local levels, using the same stress level in both cases. Global estimation evaluates the tunnel, while the local estimation differentiates each tunnel sequence. Therefore, the global prediction was calculated by taking the average of each classification system, while the local prediction was based on the evaluated quality for each tunnel sequence, using one of the systems as an input to Fig. 10 proposed by Kaiser and Moss [127].

When applying this local empirical method, it was observed that each classification system (RMR, GSI, and Q′) can generate different predictions, which may not coincide with the estimates made by other systems. Table 37 presents the estimated predictions when considering only one system, but when applying two systems, an inconsistency in the evaluation of certain tunnel sequences is observed. This difference is reflected in Table 38, where the remaining percentage corresponds to the tunnel sequences that show uncertainty in the prediction of rockburst by estimating two possible behaviors in the excavation.

**Table 38:** Percentage of matching advances when predicting the mode of behavior using two classification systems.

Systems		Behavior mode	Percentage of advances
GSI Russo [132]	RMR	M_22_	59.26%
		M_21_	1.85%
	Q’	M_22_	57.41%
		M_21_	5.56%
Cai and Kaiser [131]	RMR	M_22_	83.33%
	Q’		85.19%
Q’	RMR	M_22_	75.93%
		M_21_	1.85%

Micro-seismic monitoring has been employed in various global projects, including those in America [136], South Africa [60], Canada [137], Australia [138], and China [139]. This approach is pivotal in analyzing the spatial and temporal dynamics of failure events—both static and dynamic—during underground mining operations. Building on this foundation, we aim to apply similar techniques to a tunnel currently under study, utilizing available data such as micro-seismic records and historical failure reports. The micro-seismic data employed in this research spans from 2014 to 2017, encompassing the entire excavation period of the tunnel and extending to more than three months post-excavation.

For classifying rock burst intensity using our micro-seismic dataset, we adopted the methodology proposed by Chen et al. [140]. They categorized rock burst intensity into five distinct classes based on the radial energy observed in 133 rock bursts at the Jinping II Hydropower Station in Sichuan Province, China. This classification system provides a nuanced understanding of the seismic activity associated with the tunnel excavation and offers valuable insights for future mining and geological studies.

In this study, they developed a novel quantitative classification method for rock burst intensity, leveraging the hierarchical clustering analysis technique with the complete-linkage method, focusing on energy as the key evaluation index. The methodology introduced a refined set of criteria for classifying rock burst intensity into five distinct levels: extremely intense, intense, moderate, weak, and none. This classification is based on both the radiated energy, represented by the common logarithms of the energy levels, and the severity of damage to the surrounding rock. The intensity levels are determined by specific energy thresholds.

**Fig. 12:**
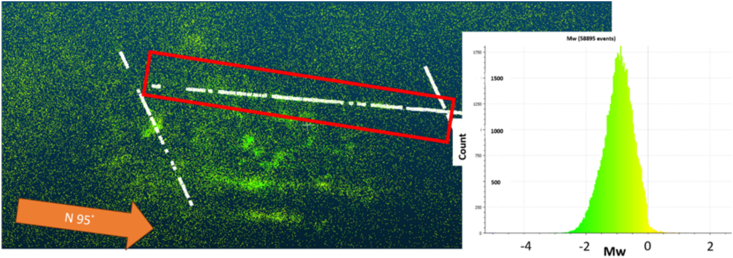
Distribution of micro-seismic data around the tunnel alongside a histogram depicting radial energy.

**Fig. 13:**
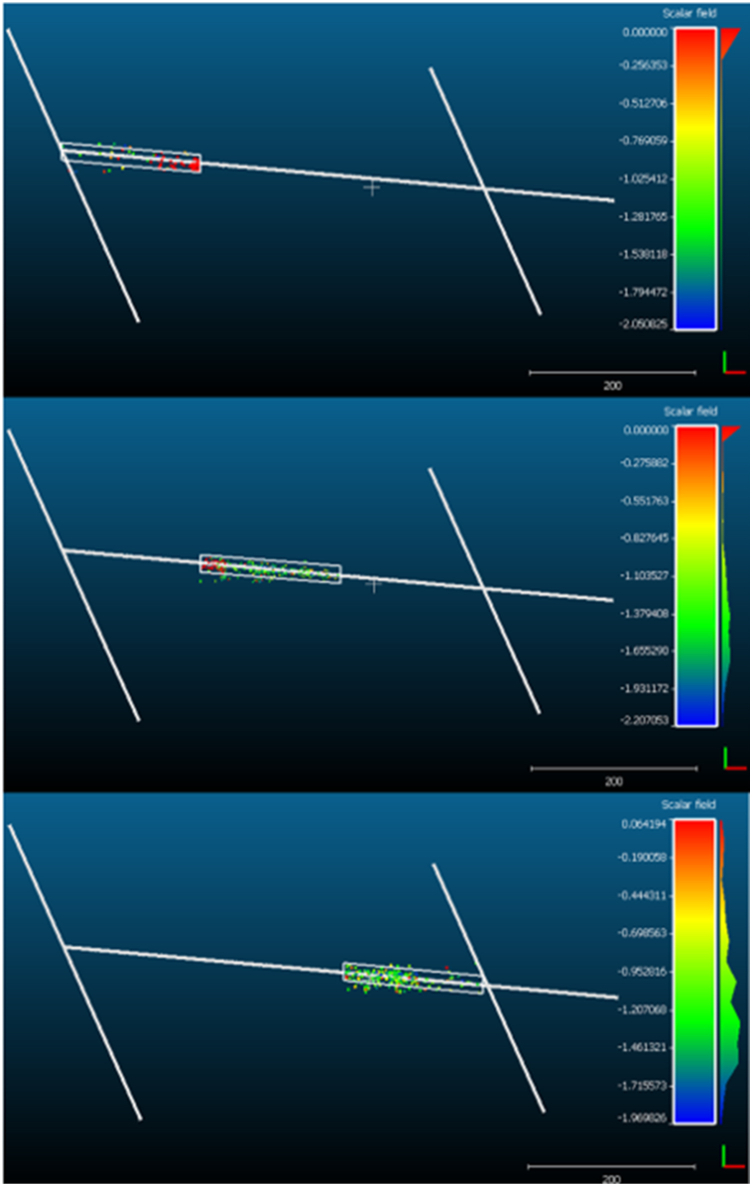
Tunnel segmented into three parts with the micro-seismic events within the specified timeframe at local scale.

Fig. 12 presents the distribution of micro-seismic data around the tunnel, alongside a histogram depicting radial energy over a five-year span from 2014 to 2017. The micro-seismic events recorded during this period, particularly from 2014 to 2017, reveal that the values are predominantly less than 2. For a more detailed analysis, the tunnel was segmented into three parts (Fig. 13), and the micro-seismic events within the specified timeframe were exclusively examined. Notably, the maximum radial energy values were observed to be less than 0, which in a logarithmic scale corresponds to the interval (-∞, 0]. According to Table 39, this interval categorizes the rock burst intensity as ‘None.'

**Table 39:** A rockburst intensity quantitative recognition criterion based on the rockburst radiated energy with rock mass failure intensity (modified after Chen and Feng [141]).

Rockburst intensities	Ig (E)	Main phenomena
None	(−∞,0]	The crack occurred inside the rock mass, an obvious failure, cannot be found on the surface of rock mass, and the cracking sound could barely be heard. No support system and construction are affected
Slight	(0,2]	Main failure type was slight spalling and slabbing in the surface of the surrounding rock mass. The rock mass was slightly ejected, and the size of ejected fragment was 10–30 cm. The cracking sound could be heard slightly, and the depth of failure was <0.5 m. If rockbolt and shotcrete lining are constructed in time, no support system and construction are damaged
Moderate	(2,4]	The main failure type was severe spalling and slabbing of the surrounding rock mass. The rock mass was obviously ejected, and the size of ejected fragment was 30–80 cm; the cracking sound was like the detonator blasting and lasted for some time inside the rock mass. The failure range was obvious, and the depth of failure was more then 0.5 m and <1.0 m. The shotcrete lining could be damaged among the rockbolts, so construction is slightly affected
Intense	(4,7]	A great deal of rock mass was suddenly ejected, and the failure range was extensive. The size of the ejected fragment was 80–150 cm, and the edge of the failure zone typically has a fresh fracture plane. A last sound could be heard before the rockburst, the rockburst, which sounded like an explosive but was louder and had an impact wave; the depth of failure was more than 1.0 m and <2.0 m. The support system was destroyed and construction were affected
Extremely intense	(7,+∞)	A large block of rock mass was suddenly ejected with and intensive seismicity, and the stability of the whole cave was seriously affected; the failure sound was like thunder or a cannonball and lasted a longer time. The depth of failure was more than 3.0 m; while the failure ranges was more extensive, the size of the ejected rock mass was greater. The support system is seriously destroyed, and construction is seriously affected

Note: Ig(E) is the common logarithm of the in situ – monitored microseismic radiated energy. The unit of E is Joule.

This observation deviates from predictions made using empirical methods, which can be attributed to several factors. Rock bursts are inherently complex phenomena influenced by multiple factors. However, most empirical approaches are based on a single-factor analysis, limiting their applicability. Additionally, these methods are often derived from limited and specific case studies, failing to encapsulate the complexity and variability inherent in each unique situation. Another significant oversight in these approaches is the neglect of the role of fractures and their infill materials, which are crucial in determining rock mass behavior. While these methods tend to provide conservative results, often predicting rock burst occurrences with varying intensities, they should be regarded as preliminary estimations, especially useful in early project phases where data is scarce. However, their application should be approached with caution due to these limitations.

## 7 Discussion

The rockburst prediction results demonstrate that the criteria applied in the New level of El Teniente mine, Chile, yield different predictions and intensities of rockburst for the same study area, leading to uncertainty in selecting the appropriate criterion to evaluate the excavation of interest. Furthermore, these criteria do not predict the exact timing or location of rockburst occurrence, resulting in a high level of uncertainty within the mine.

**Fig. 14:**
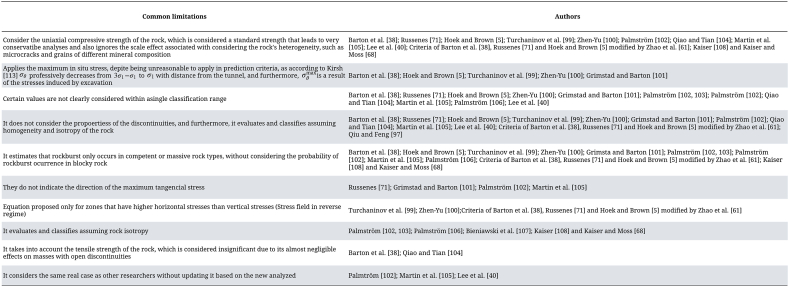
Common limitations found among the authors studied in this research.

The empirical criteria used to predict rockburst share certain common limitations, which are mentioned in Fig. 14. The most notable limitation of all empirical criteria is their potential invalidity when applied to excavations with variational boundary conditions, geological characteristics, and rock mass quality compared to the base case used to establish or evaluate the prediction criterion. Additionally, these criteria do not fully address the anisotropy of the rock. Despite these limitations, many mining companies continue to use these empirical methods due to the lack of a precise and universal criterion for accurately predicting this type of failure, which is characterized by its high complexity and difficulty of estimation.

Palmström [112] pointed out that rockburst is a failure mode caused by the behavior of the rock mass in underground openings, in terms of Terzaghi [59]. Therefore, it is important to consider the various variables that define the behavior of the ground in an underground excavation to predict the mode of instability. He et al. [65] mentioned that there are various factors that affect the occurrence of this phenomenon, such as geological conditions, excavation conditions, external disturbances, among others [60,[142], [143], [144]]. Kaiser and Cai [41], as well as Keneti and Sainsbury [145], proposed a classification of the predominant factors for the occurrence of this phenomenon, but both are based on in situ investigations and have an impact on engineering practice. For this reason, the classification made by He et al. [65], which is based on more experimental terms, was used to analyze the factors considered by each empirical method.

**Fig. 15:**
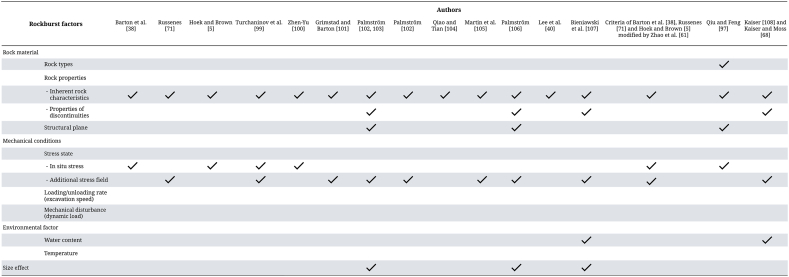
Factors influencing the occurrence of rockburst considered by the empirical methods under study.

Fig. 15 shows the parameters considered by each empirical method under study, where the criteria of Palmström [112,113], Bieniawski et al. [119], and Palmström [117] are the only ones that take into account the highest number of factors determining the occurrence of rockburst. It is important to note that the criteria of Palmström [112,113] and Palmström [117] also consider the structural plane through the structural arrangement of discontinuities, which is characterized by the shape and size of the blocks. However, the only empirical methods that consider the presence of groundwater are those proposed by Bieniawski et al. [119], Kaiser [126], and Kaiser and Moss [127], using classification systems as input parameters.

The multi-factor evaluation method proposed by Qiu and Feng [93] is the only one that considers the characteristics of geological structures, the heterogeneity of the minerals composing the rock, and the description of the environment where the underground excavation will take place allowing to consider the rock heterogeneity. Additionally, the lack of essential parameters that would allow predicting the occurrence of this phenomenon can be observed in this table. This method is the only one among those studied that considers the rock type, but it does not take into account the effect of excavation size and discontinuities on rockburst occurrence.

The most used or considered parameter for rockburst estimation is the uniaxial compressive strength of the rock, which results in very conservative analyses [95]. Another widely applied factor is the in-situ stress and the stress redistribution due to underground excavation. However, none of the empirical methods consider parameters such as temperature factor and loading/unloading rate, which are essential for understanding the occurrence of this phenomenon. Additionally, the authors did not study or consider shear failures or discontinuities and the release of dynamic disturbance waves, which are the main triggering mechanisms of rockburst in excavations [41,86,146]. It is also important to note that many empirical methods do not consider the structural plane as an input parameter, despite its relevance in tunnel engineering, as small structural planes can play an important role in rockburst [65].

From the same table (Fig. 15), it can be observed that the research demonstrated that single-index criteria do not consider all the predominant factors in rockburst, which results in these methods being unable to accurately estimate or reflect rockburst occurrence [30]. It was also evident that within these criteria, the prediction classification based on the relationship between maximum tangential stress and uniaxial strength, as well as between initial maximum principal stress and uniaxial strength, ignore the fundamental dynamic characteristics of rockburst, leading to confusion between general brittle failure and rockburst [65]. Therefore, these theories can indicate whether the studied rock mass will fail or not, but they cannot determine if that failure will be stable or unstable [147]. Additionally, both the single-index and multi-factor criteria do not consider the intermediate principal stress, which significantly influences the rock failure strength [65].

The majority of these criteria estimated that rockburst would occur throughout the entire tunnel, either with the same intensity or varying intensities, except for Palmström [112,113] and Palmström [117], which estimated that only a percentage of the tunnel would experience this type of failure. This assessment is based on input parameters that partially consider the heterogeneous property and the most influential factors that can influence the behavior of the rock mass. However, it is important to note that the used factor may not be representative due to the anisotropy of rock strength [113].

The proposed methods that allow evaluating rockburst by advance or zone, despite estimating that rockburst will occur throughout the excavation with varying intensities, are the ones by Bieniawski et al. [119], Qiu and Feng [93], Kaiser [126], and Kaiser and Moss [127]. These criteria enable predicting rockburst at both global and local levels.

**Fig. 16:**
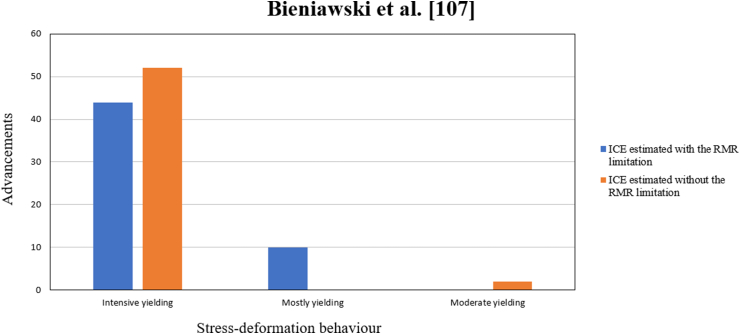
Graph of the Elastic Behavior Index (ICE) obtained by advance, considering or not considering the RMR limitation.

The criterion by Bieniawski et al. [119] presents two estimates: one considering the limitation of discontinuity sets in the RMR_89_ system and another that does not consider it, as shown in Fig. 16. When considering the number of discontinuities sets present in the zone, it can be observed that 81.4% of the advances will exhibit “intensive yielding” behavior, while the remaining percentage will have “mostly yielding” behavior. Conversely, when not considering these limitations, only 96.3% of the advances will exhibit “intensive yielding” behavior, and the remaining advances will exhibit “moderate yielding” behavior. This difference may indicate that the stress-deformation behavior will be more conservative, as well as the rock mass quality estimated by the RMR, which does not consider the quantity of discontinuity sets.

**Fig. 17:**
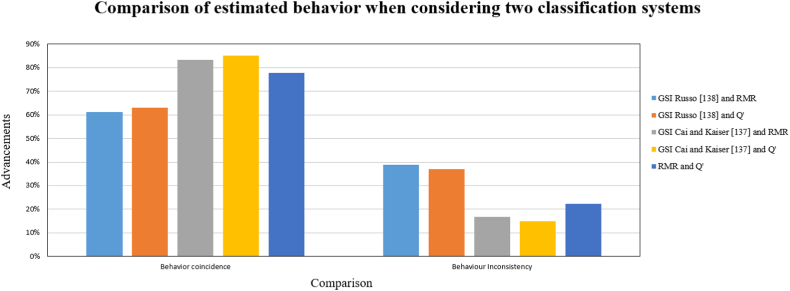
Comparative graph of the estimated behavior prediction using two classification systems.

The criterion proposed by Kaiser [126] and Kaiser and Moss [127] concluded that rockburst would occur throughout the tunnel, but with different behaviors in the local assessment. When analyzing the estimations obtained using a single classification system, it was observed that the majority predicted an M_22_ behavior mode. However, when considering two classification systems (Fig. 17), a percentage of similarity and discrepancy between the two behavior assessments were found (Table 38). The highest percentage of advances with similar behaviors was obtained when considering the GSI by Cai and Kaiser [131] compared to the other remaining systems. This similarity may be due to the fact that this new quantitative system, on a logarithmic diagram, can display a surface very close to a plane, similar to the Q′ and RMR systems [131], but with some non-linearity.

Although the RMR and Q′ systems represent the rock mass in a similar manner, one linearly and the other logarithmically [131], it was observed that 22.22% of the advances do not match the estimated behaviors. This demonstrates that when using classification systems to predict rockburst, errors in the estimations can occur.

## 8 Conclusions

In conclusion, the discussion highlights several key points regarding the prediction of rockburst in underground excavations. The criteria applied in the New level of El Teniente mine in Chile yield different predictions and intensities of rockburst for the same study area, leading to uncertainty in selecting the appropriate criterion. These criteria also do not accurately predict the timing or location of rockburst occurrence, resulting in a high level of uncertainty within the mine.

Empirical criteria used to predict rockburst have common limitations, including potential invalidity when applied to excavations with varying boundary conditions, geological characteristics, and rock mass quality. These criteria also do not fully address the anisotropy of the rock and lack precision in predicting this complex and difficult-to-estimate failure mode.

Different empirical methods consider various factors influencing rockburst occurrence, but none of them provide a comprehensive solution. Parameters such as uniaxial compressive strength, in-situ stress, and stress redistribution are commonly considered, but essential factors like loading/unloading rate, shear failures, discontinuities, and dynamic disturbance waves are often overlooked. The structural plane is also not consistently accounted for in the criteria, despite its relevance in tunnel engineering.

The research demonstrates that single-index criteria fail to consider all the predominant factors and may confuse general brittle failure with rockburst. They can determine whether the rock mass will fail but not if the failure will be stable or unstable. The intermediate principal stress, which significantly influences rock failure strength, is also not considered in most criteria. Most criteria estimate that rockburst will occur throughout the entire tunnel, either with the same intensity or varying intensities. Some criteria allow for evaluating rockburst by advance or zone, providing predictions at global and local levels. The criterion proposed by Bieniawski et al. [119] considers the limitation of discontinuity sets in the Rock Mass Rating (RMR) system and shows variations in stress-deformation behavior. Kaiser [126] and Kaiser and Moss [127] criteria predict rockburst throughout the tunnel but exhibit discrepancies in local assessments when using different classification systems.

The discussion emphasizes the limitations and uncertainties associated with current empirical criteria for rockburst prediction. While these criteria consider various factors, they lack a precise and universal criterion to accurately predict rockburst occurrence. Further research and advancements are needed to develop more comprehensive and reliable methods for rockburst prediction in underground excavations.

While these methods tend to provide conservative results, often predicting rock burst occurrences with varying intensities, they should be regarded as preliminary estimations, especially useful in early project phases where data is scarce. However, their application should be approached with caution due to these limitations.

## CRediT authorship contribution statement

**Nayadeth Cortés:** Writing – review & editing, Writing – original draft, Visualization, Validation, Methodology, Investigation. **Amin Hekmatnejad:** Writing – review & editing, Writing – original draft, Visualization, Validation, Supervision, Software, Resources, Project administration, Methodology, Investigation, Funding acquisition, Formal analysis, Data curation, Conceptualization. **Pengzhi Pan:** Validation, Methodology, Investigation, Formal analysis. **Ehsan Mohtarami:** Writing – review & editing, Validation, Investigation. **Alvaro Pena:** Writing – review & editing, Validation, Investigation. **Abbas Taheri:** Writing – review & editing, Validation, Methodology, Conceptualization. **Cristian González:** Validation, Formal analysis, Data curation.

## Declaration of competing interest

The authors declare the following financial interests/personal relationships which may be considered as potential competing interests: Amin Hekmatnejad reports financial support was provided by National Agency for Research and Development. No If there are other authors, they declare that they have no known competing financial interests or personal relationships that could have appeared to influence the work reported in this paper.
